# Dynamics of street environmental features and emotional responses in urban areas: implications for public health and sustainable development

**DOI:** 10.3389/fpubh.2025.1589183

**Published:** 2025-06-24

**Authors:** Yangfei Huang, Chenjian Zhong, Tangtao He, Yuyang Jiang

**Affiliations:** ^1^School of Civil Engineering & Architecture, Zhejiang University of Science & Technology, Hangzhou, China; ^2^Center of Urban and Rural Development, Zhejiang University of Science & Technology, Hangzhou, China; ^3^Zhejiang-Singapore Joint Laboratory for Urban Renewal and Future City, Zhejiang University of Science & Technology, Hangzhou, China; ^4^College of Architecture and Urban Planning, Guangzhou University, Guangzhou, China; ^5^School of Geography and Planning, Cardiff University, Cardiff, United Kingdom

**Keywords:** green space, street view, Green View Index, machine learning, emotional responses, public health, health planning, sustainable development

## Abstract

**Introduce:**

Urban street spatial quality, as an intervenable environmental factor from the perspective of public health, significantly affects residents' mental health and emotional wellbeing. Accurately identifying emotional hot spots in urban environment and exploring the mechanism of environmental features affecting emotions are crucial for improving residents' mental health level, promoting healthy urban planning and creating a sustainable urban environment.

**Methods:**

This study employed an interdisciplinary approach, utilizing street view images from Liwan District, Guangzhou, China. A Pyramid Scene Parsing Network (PSPNet) was applied to quantify 18 key environmental features, including the Green View Index (GVI), Space Openness (SO), Enclosure Index (EI), etc. By integrating an emotion dataset assessed by 40 experts, a random forest model was constructed to predict emotional responses to different street spaces. Emotional distribution maps were generated using ArcGIS Pro to identify emotional hotspots. Subsequently, SHAP (SHapley Additive exPlanations) analysis was conducted to explore how environmental features influence emotional responses.

**Results:**

The analysis revealed the following: (1) Positive emotions were significantly associated with areas of well-vegetated, while negative emotions were predominantly concentrated in industrial zones and narrow alleys. (2) GVI, sky-green ratio, EI, and SO had a notable impact on emotional responses. (3) The optimal range for the GVI (0.27–0.3) was found to maximize positive emotional valence. Beyond this range, further increases in the GVI did not result in significant emotional changes.

**Discussion:**

This study demonstrates the feasibility of predicting public emotional responses from street view images using machine learning. Optimizing green spaces and improving pedestrian environments can promote emotional health. To effectively balance the distribution of urban green spaces and maximize public health benefits, it is recommended that governments collaborate with communities, leveraging fiscal incentives and green infrastructure investments to promote equitable and sustainable development of green spaces. These findings play a crucial role in advancing both public health and environmental sustainability.

## 1 Introduction

From the perspective of health geography, urban street space, as an active element in public health intervention, is undergoing a shift in values—from prioritizing efficiency to emphasizing human wellbeing. To optimize street space, it is crucial to improve its potential for environmental healing through designs that provide emotional support. This approach not only helps improve residents' mental health but also addresses disparities in social welfare, providing spatial solutions for health equity. By adopting design strategies that foster positive emotional experiences, the street environment can be optimized to enhance residents' sense of wellbeing and contribute to the creation of more psychologically supportive urban spaces (1). By adopting design strategies that promote positive emotional experiences, the street environment can be optimized to enhance residents' wellbeing and contribute to the creation of more psychologically supportive urban spaces. Various countries and regions, such as those in Scandinavia and the United States, have implemented measures to optimize street spaces, focusing on the introduction of green spaces and enhancing walkability to foster residents' mental health and social interactions (2, 3). However, existing urban governance frameworks often neglect the emotional aspect of place-making, potentially exacerbating health inequalities among different socio-economic groups. For example, computer vision assessments have revealed stark disparities in neighborhood visual environments along socio-economic lines (4), underscoring that poorer communities often endure inferior streetscape conditions. Recent research also advocates leveraging artificial intelligence to analyze the built environment for public health, aiming to uncover and address such spatial inequities (5). As such, the deep connection between street environments and residents' emotional perceptions warrants urgent exploration. This will provide theoretical support for the creation of high-quality street spaces and promote the development of cities in a more livable and human-centered direction.

Emotion serves as a bridge between humans and their environments, reflecting subjective perceptions of space and influencing behavior and social relationships (6–8). The AR model provides a theoretical framework for the interaction between environmental perception, emotional responses, and behavior, suggesting that environmental features influence emotions through perception, with emotions playing a crucial mediating role in this process (9). Therefore, the design of street environments should not only meet physical functional needs but also consider their potential impact on residents' emotional states. Quantitative monitoring of emotional states, as a new perspective in public health research, with its multidimensional features (such as pleasure, anxiety, etc.) (10), can not only effectively assess the quality of the built environment but also provide valuable insights for preventive public health interventions (11, 12). Nevertheless, studies have revealed biases when comparing objective mappings of urban environments with residents' subjective perceptions (13), highlighting that conventional assessments may overlook the human experiential dimension. To bridge this gap, recent approaches have begun integrating multi-source urban data—combining street-view imagery, real-time traffic patterns, points of interest, and survey-based comfort evaluations—to assess pedestrian experiences more holistically (14).

Visual perception, as a core dimension of urban planning, is predominantly reflected in the direct impact of visual indicators such as the Green View Index (GVI) on emotional responses (15). The influence of street spatial features on residents' emotional perceptions has gradually become a research focus in recent years (16, 17). Various studies on street environments have proposed evaluation indicators to characterize the material features and quality of street spaces, providing a foundation for understanding the complex relationship between street environments and emotional responses. The GVI, and space openness (SO) are core indicators for assessing street greenery and visual openness (18–21). In addition, scholars such as Jacobs (22) and Jan Gehl (23) have emphasized that street design should prioritize walkability over vehicle flow efficiency, as high-density traffic and narrow passageways are considered major factors that undermine pedestrians' sense of safety (24, 25). Epidemiological studies have confirmed that exposure to high traffic volume is significantly positively correlated with pedestrian anxiety levels, while walkability-oriented design has been shown to reduce the risk of cardiovascular diseases (26, 27). Furthermore, pedestrian presence, as a key indicator of street vitality, not only reflects the degree of support the street offers for walking activities but also plays an important role in enhancing residents' positive emotional perceptions. The diversity of architectural and landscape features (such as facade decorations) can enhance visual complexity, potentially facilitating human-environment interaction (28, 29). Finally, based on the principles of diversity and comprehensiveness, we considered various aspects such as spatial physical features, visual perception, and accessibility, and selected 18 indicators, including GVI, SO, Accessibility, Enclosure Index (EI), and Complexity, as means to evaluate the features of street environments.

The research focuses on six emotions: pleasure, relaxation, curiosity, anxiety, unsafety, and loneliness. Their selection was informed by prior empirical studies and established emotion theories. Akgün-Tanbay et al. (30) and Bivina and Parida (31) highlight that pedestrians prioritize safety and comfort, with emotions such as relaxation, pleasure, and safety directly reflecting the quality of the walking experience (32). Specifically, pleasure corresponds to positive emotions evoked by aesthetically pleasing, comfortable, and green environments, aligning with the pleasure dimension in Russell's widely used pleasure–arousal–dominance (PAD) framework (33). Relaxation draws on Kaplan's (34) Attention Restoration Theory (ART), which posits that restorative urban features (e.g., greenery, open spaces) alleviate mental fatigue and promote calm. Curiosity, often expressed as attraction, reflects the likelihood of pedestrians choosing a particular street (35). Anxiety and unsafety reflect psychological distress and perceived threats in public spaces, key factors in evaluating pedestrian wellbeing (36). Loneliness captures perceived social disconnection within urban environments, strongly associated with public health concerns such as depression and isolation (36). Collectively, these six emotions provide a balanced representation of positive and negative experiences, and cover hedonic, safety-related, cognitive, and social dimensions of pedestrians' subjective perceptions of the urban environment. Their spatial distribution features can provide decision-making insights for the allocation of community mental health services and resources.

Street view big data captures the street environment at the human visual scale using panoramic images, providing a spatially organized, high-precision data foundation for quantifying emotional perception. Its integration with computer vision technologies, such as semantic segmentation, has significantly enhanced the efficiency of identifying environmental features. High-performance algorithms, such as the Mask R-CNN developed by He et al. (37) and the Pyramid Scene Parsing Network (PSPNet) proposed by Zhao's team (38), have greatly improved the accuracy of identifying urban environmental elements (39). These advancements provide a technological foundation for large-scale urban spatial quantification research and drive the paradigm shift in public health research from traditional epidemiological surveys to Digital Health, aligning with the technological innovation focus of the WHO Healthy Cities assessment. The street scene perception model developed by Zhang's team (39) using the PlacePulse dataset enables macro-scale urban perception evaluation. Qi et al. (40) revealed the spatial distribution mechanisms of street vitality by extracting visual features through deep neural networks and proposed environmental optimization strategies. While existing research has confirmed the effectiveness of street view data in environmental analysis (41–45), challenges remain in the analysis of emotional perception mechanisms. First, many previous studies have used linear regression models (e.g., OLS) to examine the impact of the environment on emotions, which oversimplifies the complex interactions between environmental features and emotional responses (46–49), making it difficult to capture threshold effects of environmental features like the GVI and nonlinear responses. Even advanced data-driven studies tend to rely on linear correlations for environment–perception relationships (50), indicating that key nonlinear dynamics remain unaddressed. Second, these studies have neglected the spatial heterogeneity of emotional geography and failed to systematically explore the patterns of emotional distribution, resulting in a lack of spatial targeting in intervention designs.

To address these limitations, this study integrates Explainable Artificial Intelligence (XAI) with the One *Health* framework, responding to the WHO's initiative on the relationship between the built environment and mental health. An interdisciplinary analytical framework is developed, combining Geographic Information Systems (GIS) to quantify spatial heterogeneity, SHAP attribution analysis to decipher nonlinear interaction pathways, and deep coupling of psychological emotional dimensions with computer vision-based feature extraction. This framework advances beyond single-disciplinary paradigms with a methodological innovation for emotion-responsive street design, enabling targeted identification of emotional hotspots as spatial clusters of predicted intensity and revelation of differentiated thresholds for features like GVI.

The study focuses on the Liwan District of Guangzhou, Guangdong Province, China. As one of Guangzhou's historical urban cores, Liwan District possesses rich cultural heritage and diverse street morphologies, making it a representative and highly relevant case study. Using the PSPNet technique, the study performs semantic segmentation on street-view images to extract urban environmental features. Based on these features, a random forest algorithm is employed to construct an emotional perception prediction model, capturing six emotions: pleasure, relaxation, curiosity, anxiety, unsafety, and loneliness. This study uses SHAP attribution analysis to explore the potential nonlinear interactions between street environment features and residents' emotional responses. By leveraging street-view big data and machine learning techniques, it introduces an innovative multidimensional framework for measuring and analyzing emotional perceptions. This framework provides technological support for urban perception research and enhances the understanding of how street environments affect human emotions.

The study aims to achieve the following three objectives:

(1) To construct a multidimensional emotional perception prediction model based on street-view images from the Liwan District;
(2) To predict and reveal the emotional geography features of Liwan District, clarifying the distribution patterns of emotions in street spaces;
(3) To explore the complex nonlinear relationship between street environment features and residents' emotional perceptions using SHAP attribution analysis.

In response to the growing global burden of mental health issues, this research combines street-view big data with Explainable Artificial Intelligence (XAI) to analyze the nonlinear impact of street environments on emotional perception. By identifying the spatial distribution patterns of emotional hotspots and their associated environmental features, the study provides strong support for urban design strategies and offers evidence-based spatial intervention solutions for policymakers in the development of healthy cities. This research aims to help reduce the mental health burden caused by environmental factors and contribute to the advancement of urban health research.

## 2 Materials and methods

### 2.1 Study area

**Figure 1:**
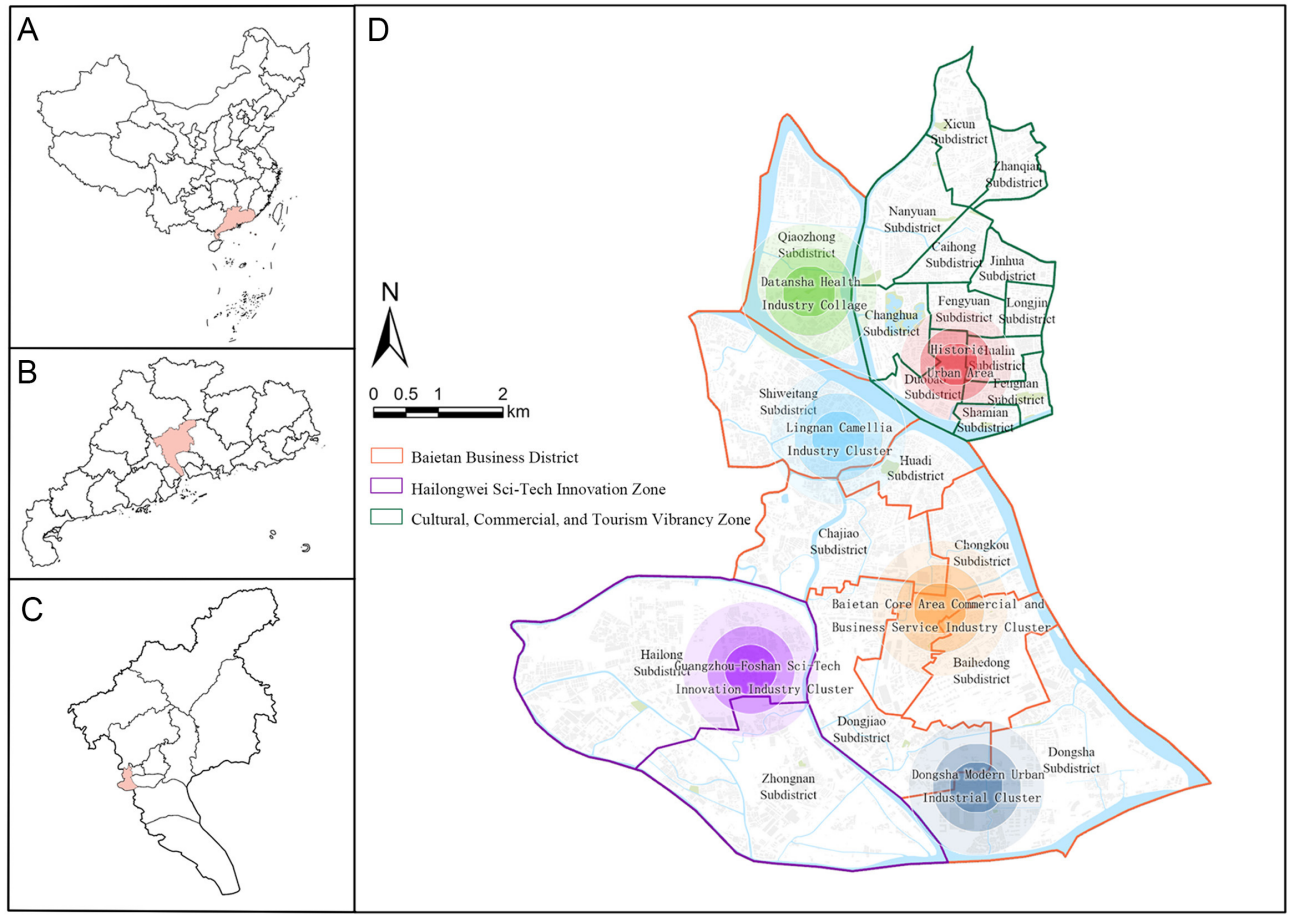
Study area. **(A)** China, **(B)** Guangdong province, **(C)** Guangzhou city, **(D)** Liwan District.

The study focuses on Liwan District, Guangzhou, China (23°07′ N, 113°15′ E; population: 1.12 million), is a dense urban area with mixed land-use and varied street patterns, reflecting typical characteristics of rapidly urbanizing cities in southern China (Figure 1). Recent initiatives inspired by the WHO's “Healthy Cities” framework have focused on improving the built environment through green space development and pedestrian infrastructure upgrades (51), The availability of high-resolution street view data facilitates detailed analysis of micro-scale environmental features and their associations with health disparities identified in previous research (52).

### 2.2 Data sources and data collection

#### 2.2.1 Street view image data collection

**Figure 2:**
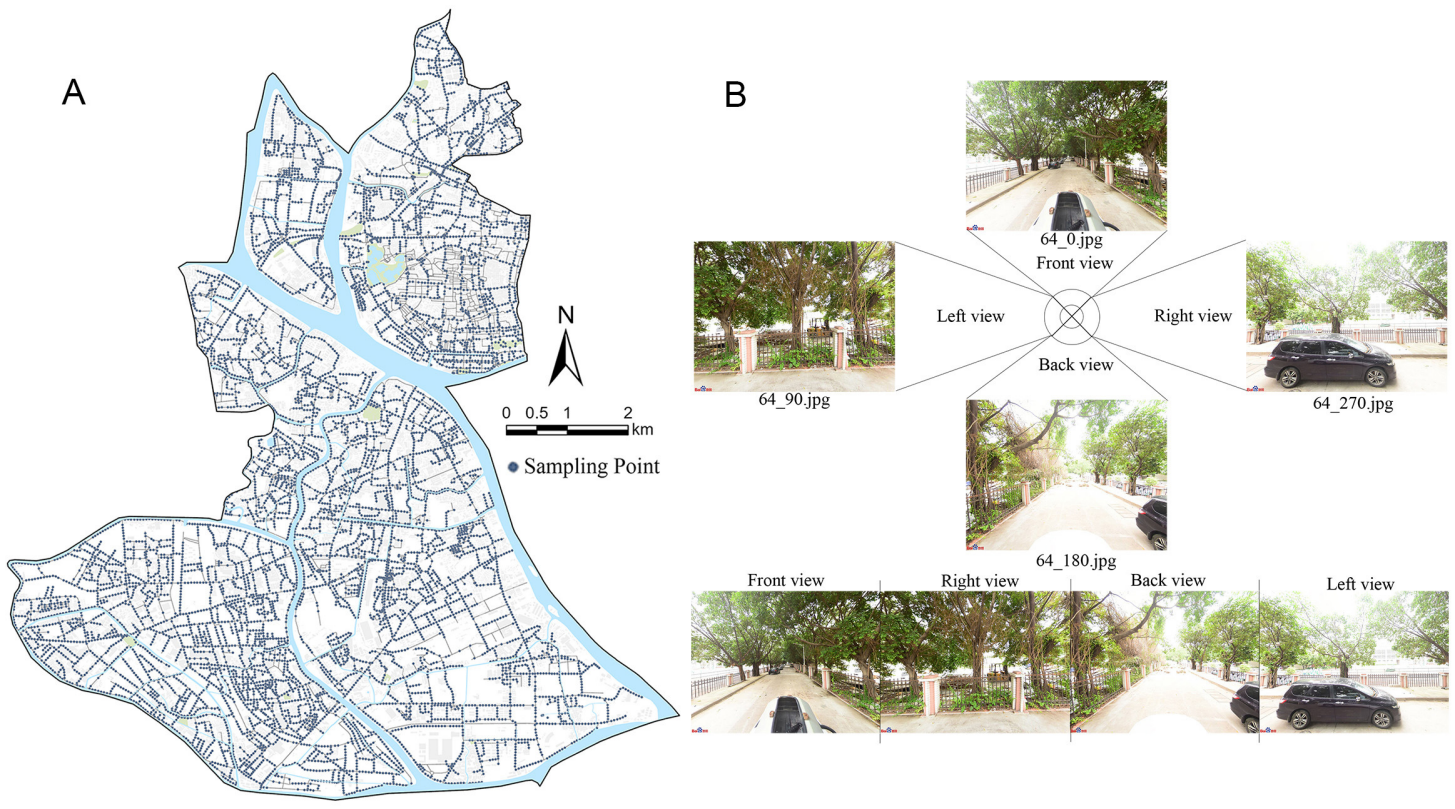
Street attractions sampling map. **(A)** Road network and its sampling points, **(B)** street panorama composed of street view from four directions.

This study utilizes the Baidu Maps Static API to scrape street-view image data. Road network data is extracted and optimized from OpenStreetMap (Remove highways and add sidewalks), resulting in a corrected road centerline with a total length of 619,073 meters. Using ArcGIS Pro software, sampling points are set at 50-meter intervals along the road centerline, with 11,911 WGS1984 geographic reference coordinates selected (Figure 2A). To enhance efficiency and reduce environmental impact, Python 3.7 is used to automatically obtain images from the Baidu Maps API. Four street-view images (1,000 × 750 pixels) are captured from each sampling point in four directions (Figure 2B). The final dataset consists of 9,955 valid sampling points and 39,820 high-resolution images, covering a variety of environments to ensure the representativeness of the sampling.

#### 2.2.2 Emotional response training data acquisition

Expert elicitation was conducted to establish reliable emotional response metrics. A multidisciplinary panel of 40 experts, including urban planners, psychologists, and public health specialists, evaluated 600 randomly selected street view images using a five-point Likert scale. Emphasis was placed on six key emotional dimensions: pleasure, relaxation, curiosity, anxiety, unsafety, and loneliness. Structured consensus discussions resolved inter-rater discrepancies, ensuring consistent and validated emotional ratings. Final scores were categorized into three tiers: (1) negligible, (2) moderate, and (3) significant.

### 2.3 Methodology

**Figure 3:**
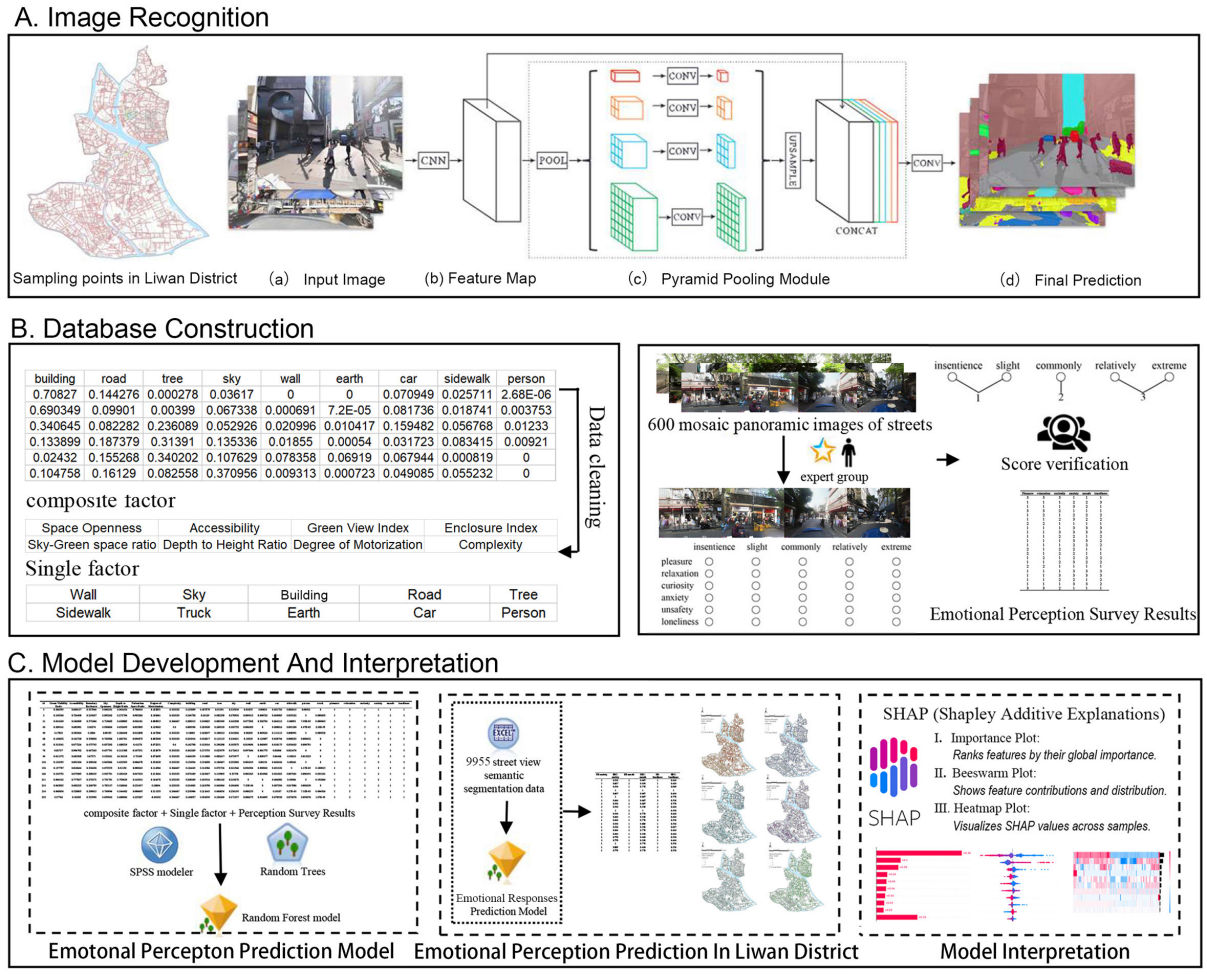
Research workflow for modeling emotional responses to urban street environments. **(A)** Image recognition, **(B)** database construction, **(C)** model development and interpretation.

The study consists of three primary phases: image recognition, database construction, and model development and interpretation. Figure 3 illustrates the overall process of studying emotional responses in urban street spaces using big data and machine learning.

Image recognition phase: Street View images were collected from sampling points, and a PSPNet deep learning model was applied for semantic segmentation to extract the pixel proportions of features in the Street View. This method enhanced the accuracy of environmental feature extraction while reducing reliance on manual surveys, fostering sustainable research.

Database construction phase: Eighteen key indicators, encompassing both composite and individual metrics, were derived from the PSPNet segmentation results to construct the spatial environmental feature database. An emotional response database was also developed based on expert ratings.

Model development and interpretation phase: A random forest model was developed using SPSS Modeler for predictions, while ArcGIS Pro was used to generate layered emotional response maps. To interpret the model, SHAP (Shapley Additive Explanations) was applied, providing insights into how different features contributed to the predictions. This approach allows for a deeper understanding of the complex, nonlinear relationships between environmental features and emotional responses, helping urban planners prioritize interventions that improve sustainability and wellbeing.

#### 2.3.1 Extraction of street environment features

This study used PSPNet for semantic segmentation of street view images. PSPNet, with its Pyramid Pooling Module (PPM), performs multi–scale feature extraction and fusion, capturing global scene context. This reduces misidentification of fine–grained features in complex urban areas and improves discrimination of spatial elements, demonstrating its effectiveness in analyzing intricate urban scenes.

Trained on the ADE20K dataset, the PSPNet model achieved a mean Intersection over Union (mIoU) over 80%, indicating great performance in multi–object semantic segmentation tasks (38). It's ideal for analyzing diverse street view environmental features. Using PSPNet, we segmented street view image elements and extracted key features such as GVI, building coverage, and SO, generating a high–resolution spatial feature dataset.

By integrating multi-scale feature extraction with semantic segmentation, PSPNet enables precise quantification of the pixel proportions of street view features, providing high-quality data to explore the relationship between street environments and emotional response.

#### 2.3.2 Construction of environmental features indicators

Based on the results of semantic segmentation, this study developed a series of environmental feature indicators encompassing both single and composite features. Single features include the pixel proportions of roads, buildings, vegetation, and sky, while composite indicators, such as GVI, SO, EI, and spatial complexity, provide more detailed representations of the urban environment (Table 1). These indicators comprehensively reflect the physical features of street environments and play a critical role in quantifying residents' emotional response.

**Table 1:** Components of composite indices.

**Indicator**	**Description**	**Objective function equation**
Green View Index	The proportion of pixels representing vegetation and grass in the street scene.	$C_{1}=\frac{P_{tree}+P_{grass}+P_{plant}}{P_{all}}$
Accessibility	The ratio of vehicle pixels to total road pixels in the street view image, used as a measure of road traffic flow.	$C_{2}=1-\frac{P_{car}}{P_{car}+P_{road}}$
Enclosure Index	The degree of spatial enclosure created by buildings, trees, and vertical structures (walls, signboards, streetlights).	$C_{3}=\frac{P_{building}+P_{tree}+P_{struct}}{P_{all}}$
Space Openness	The proportion of open space, excluding surrounding building masses, in the street view image.	$C_{4}=1-\frac{P_{building}}{P_{all}}$
Depth to height ratio	The ratio of the average height of buildings on both sides of the street to the street width.	$C_{5}=10.459e^{-5.2094P_{sky}}$
Degree of motorization	The proportion of street space occupied by motor vehicle lanes.	$C_{6}=\frac{P_{road}-P_{sidewalk}-P_{path}-P_{runway}}{P_{all}}$
Complexity	The ratio of the number of street elements with a proportion exceeding 0.1%−30%.	$C_{7}=\frac{Count_{p}}{30}$
Sky-green space ratio	The proportion of sky area and vegetation area in the image.	$C_{8}=\frac{P_{tree}+P_{grass}+P_{plant}+P_{sky}}{P_{all}}$

^**^P_category_ represents the proportion of the category in the image.

#### 2.3.3 Development and interpretation of the emotional response prediction model

This study utilized SPSS 27.0 for descriptive analysis and IBM SPSS Modeler 18.0 to develop a random forest model. Descriptive statistics analyzed the frequency distribution and trend changes of the data. The random forest algorithm, proposed by Breiman (53), is an ensemble learning method based on decision trees, characterized by high prediction accuracy and robust tolerance to outliers and noise. By constructing many decision trees and aggregating their outputs, the random forest algorithm improves performance, making it suitable for large datasets with high efficiency and strong predictive power.

In this study, the random forest regression model used street spatial features to predict residents' emotional response scores, assessing the influence of spatial features on emotional responses.

To address the random forest model's “black-box” nature and enhance interpretability of feature contributions, the study used SHAP (54), a game theory-based method that quantifies each feature's marginal impact on model predictions. SHAP not only captures complex nonlinear associations between variables, but also enables a quantitative assessment of feature importance, thereby providing a novel framework for elucidating the latent mechanisms linking emotional responses with street environment characteristics. It supports both global understanding and local interpretability by revealing how individual features influence specific predictions.

As an additive model, SHAP treats each feature as a contributor, calculates its contribution, and sums the contributions to generate the final prediction. In this study, SHAP values were the interpretation tool. The random forest model was configured with key hyperparameters, including the minimum samples at each leaf node (min_samples_leaf = 5) and the number of trees (n_estimators = 200). SHAP values for the test set samples were calculated by instantiating shap. Kernel Explainer with the prediction function and the training dataset. The specific calculation process of SHAP is as follows (55):


$$\begin{array}{r} SHAP_{j}=\underset{S\subseteq \left\{V_{1},V_{2},...,V_{p}\right\}\backslash \left\{V_{j}\right\}}{\sum }\;\frac{|S|!\left(p-|S|-1\right)!}{P!}\\ \left[f_{x}\left(S\cup \left\{V_{j}\right\}\right)-f_{x}\left(S\right)\right]\\ y_{i}=y_{base}+\overset{k}{\underset{j=1}{\sum SHAP\left(x_{ij}\right)}} \end{array}$$

In the formula: *SHAP*_*j*_ is the *SHAP* value of any sample in feature *j*; S is the subset of features used in the model; *V*_*p*_ is the set of features in the model; *p* is the number of features; *f*_*x*_(*S*) is the prediction result of the model in the feature subset; *y*_*i*_ is the prediction result at sample *i*; *y*_*base*_ is the average value of the prediction values of other samples; *SHAP*(*x*_*ij*_) is the *SHAP* value of sample i at feature *j*; k is the number of features. The *SHAP* value is an additional feature attribution method, which interprets the prediction value of the model as the sum of the attribution values of each input feature. Therefore, the positive and negative values of *SHAP*(*x*_*ij*_) express the specific impacts of different street view space features on emotional response.

By integrating high-precision street view data, deep learning-based semantic segmentation, and the *SHAP* method, this study achieved a scientifically rigorous full-chain approach from data collection to result interpretation. Moreover, the developed methodological framework demonstrates strong adaptability, enabling its extension to other high-density urban environments and providing feasible solutions for achieving sustainable urban development.

## 3 Results

### 3.1 Description and analysis of the built environment

The study conducted a multi-dimensional characteristic analysis of the built environment data, aiming to comprehensively understand the influence mechanism of various elements of the built environment on residents' emotions. Table 2 presents the descriptive statistics of each indicator. For example, the mean value of the GVI is 0.175, indicating a relatively low level of greenery. The mean value of the accessibility indicator is 0.696, reflecting a high level of accessibility in the region, which enables residents to easily access amenities and services. These metrics, combined with the degree of motorization and environmental complexity, elucidate urban emotional dynamics.

**Table 2:** Descriptive statistics of built environment.

**Sort**	**Factor**	**Min**	**Max**	**Mean**	**SD**
1	Green View Index	0.000000	0.619938	0.175172	0.137401
2	Accessibility	0.000000	0.959919	0.695675	0.186783
3	Enclosure Index	0.073105	0.922809	0.447119	0.145424
4	Space Openness	0.154934	0.999366	0.749251	0.172771
5	Depth to height ratio	0.000082	1.000000	0.227872	0.267498
6	Degree of motorization	−0.268076	0.360933	0.129168	0.095299
7	Complexity	0.166667	0.600000	0.335278	0.069004
8	Sky-green space ratio	0.001602	0.831097	0.336281	0.164879
9	Building	0.000828	0.473067	0.159009	0.105615
10	Road	0.000634	0.845066	0.250889	0.172005
11	Tree	0.001381	0.379706	0.170486	0.076613
12	Sky	0.000000	0.514718	0.143538	0.123806
13	Wall	0.000000	0.621462	0.032476	0.063889
14	Earth	0.000000	0.302734	0.020398	0.039373
15	Car	0.010028	0.316441	0.082067	0.049900
16	Sidewalk	0.000000	0.325158	0.036644	0.044324
17	Person	0.000000	0.093076	0.002551	0.007126
18	Truck	0.000000	0.414443	0.011383	0.041960

### 3.2 Analysis of emotional response model prediction results

The training results are presented in Table 3. The model aligns with subjective evaluations, identifying intervention hotspots (e.g., green space development). For example, areas with low greenery and high anxiety levels replace with require vegetation augmentation and micro-green space implementation.

**Table 3:** Model accuracy of random forest classifiers.

**Dataset type**	**Pleasure**	**Relaxation**	**Curiosity**	**Anxiety**	**Unsafety**	**Loneliness**
Training set	97.22%	95.51%	91.24%	97.22%	94.87%	95.30%
Test set	75.76%	71.97%	55.30%	72.73%	69.70%	65.91%

Using the trained random forest model, we generated emotional response predictions for the streets (Table 4). Results show higher levels of pleasure (mean value 1.96, with 22.1% experiencing high levels), indicating that residents generally feel positive about their street environments. However, loneliness and unsafety were lower (mean 1.35 and 1.33, respectively), with only 7.5 and 2.1% reporting high levels. This suggests a need to focus on improving street spaces to reduce negative emotions. Relaxation was relatively common (mean 1.71, 10.5% high), but curiosity was weaker (mean 1.49, 29.3% moderate). Anxiety was moderate (mean 1.57).

**Table 4:** Emotional responses predictions (Liwan District).

**Emotion**	**Mean value**	**Low (%)**	**Medium (%)**	**High (%)**	**Low perception**	**Medium perception**	**High perception**
Pleasure	1.96	26.4	51.5	22.1	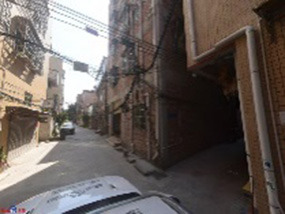	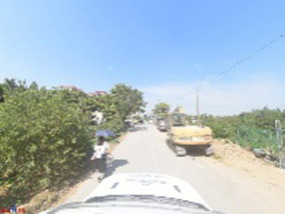	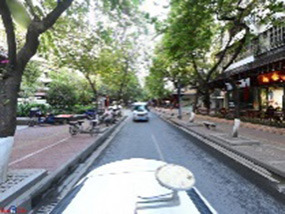
Relaxation	1.71	39.6	49.9	10.5	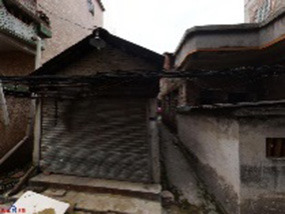	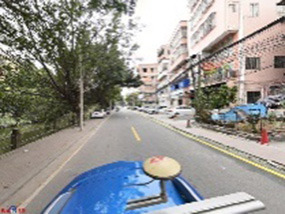	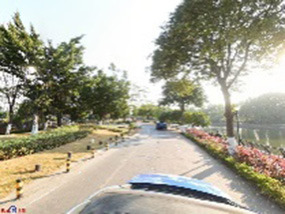
Curiosity	1.49	61.0	29.3	9.7	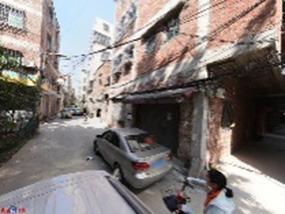	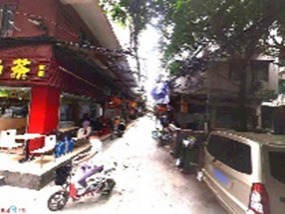	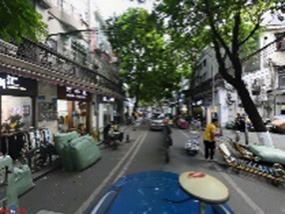
Anxiety	1.57	51.6	40.3	8.1	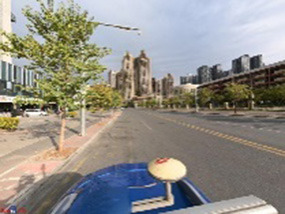	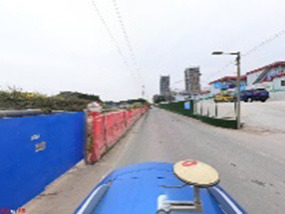	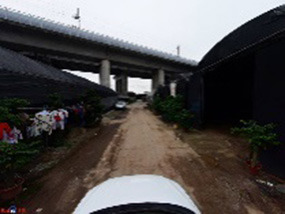
Unsafety	1.33	69.0	29.0	2.1	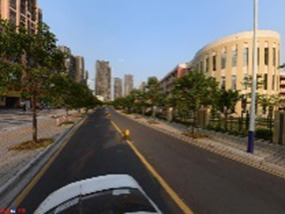	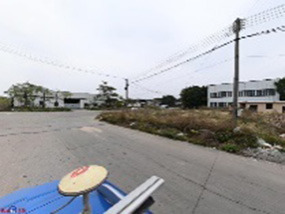	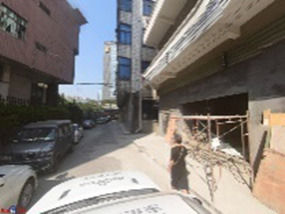
Loneliness	1.35	71.1	22.4	7.5	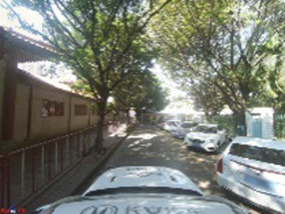	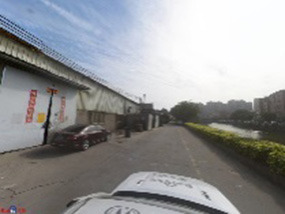	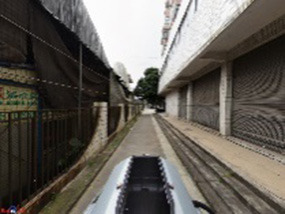

### 3.3 Geospatial analysis of emotional perception

The Pearson correlation analysis of six emotional responses (Table 5) identified significant correlations (*p* < 0.01) among most emotions, except between loneliness and curiosity. Positive emotions (pleasure, relaxation, curiosity) exhibited strong intercorrelations, with pleasure-relaxation (*r* = 0.522), relaxation-curiosity (*r* = 0.48), and pleasure-curiosity (*r* = 0.55). Negative emotions (anxiety, unsafety, loneliness) also correlated positively, including anxiety-unsafety (*r* = 0.477), unsafety-loneliness (*r* = 0.139), and anxiety-loneliness (*r* = 0.173). Spatial trend consistency across emotional categories reflects urban features' universal perceptual influence. Notably, the strong association between pleasure and relaxation (*r* = 0.522) suggests that enhancing relaxation-promoting elements (e.g., green spaces, reduced congestion) can significantly improve overall emotional wellbeing.

**Table 5:** Correlations between environmental features and emotional responses.

**Emotion**	**Pleasure**	**Relaxation**	**Curiosity**	**Anxiety**	**Unsafety**	**Loneliness**
Pleasure	1	0.522^**^	0.055^**^	−0.488^**^	−0.384^**^	−0.137^**^
Relaxation	0.522^**^	1	0.048^**^	−0.511^**^	−0.405^**^	−0.149^**^
Curiosity	0.055^**^	0.048^**^	1	−0.026^**^	−0.047^**^	0.032^**^
Anxiety	−0.488^**^	−0.511^**^	−0.026^**^	1	0.477^**^	0.173^**^
unsafety	−0.384^**^	−0.405^**^	−0.047^**^	0.477^**^	1	0.139^**^
Loneliness	−0.137^**^	−0.149^**^	0.032^**^	0.173^**^	0.139^**^	1

** At the 0.01 level (two-tailed), the correlation is significant.

**Figure 4:**
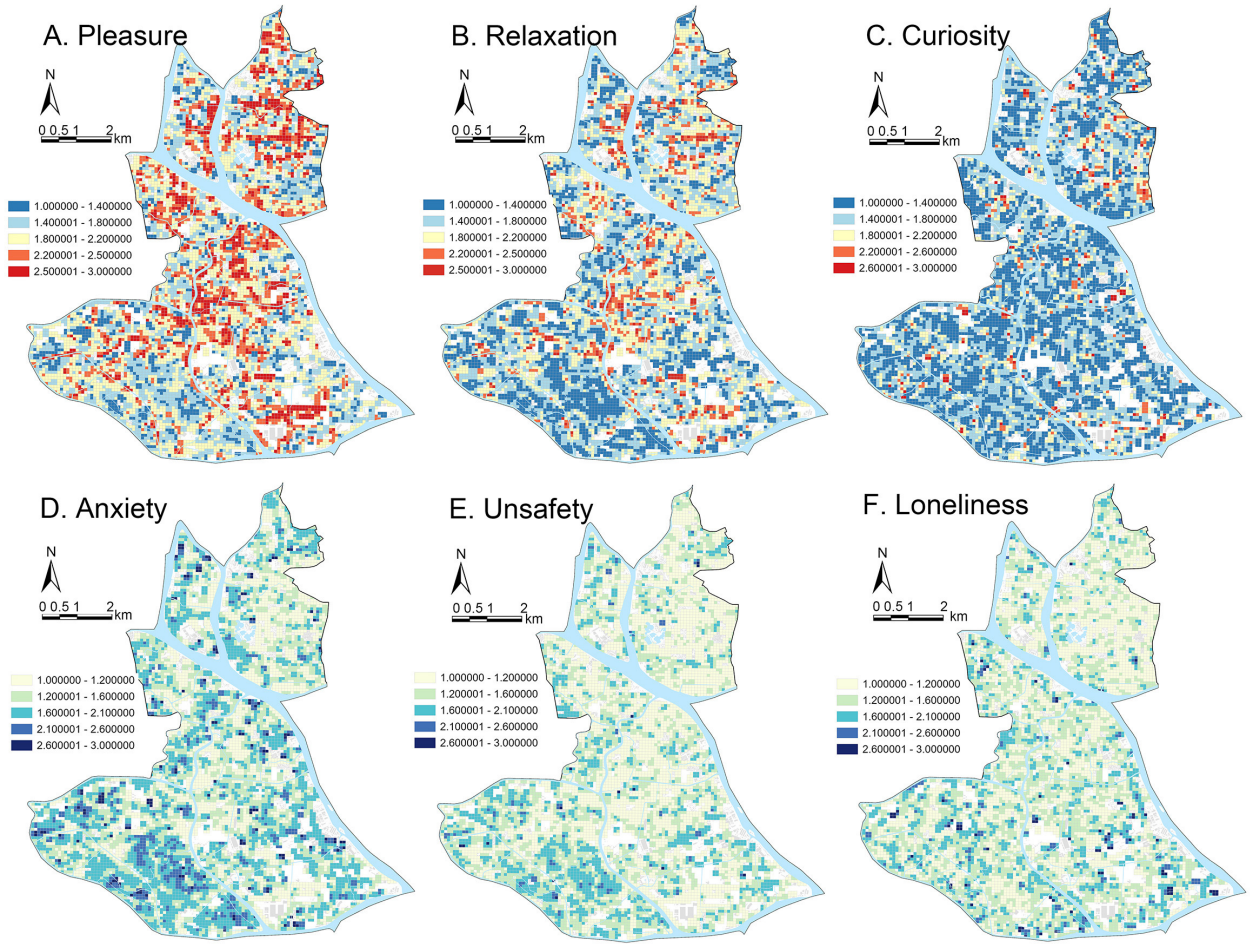
Spatial distribution of predicted emotional responses in Liwan District. **(A)** Pleasure, **(B)** relaxation, **(C)** curiosity, **(D)** anxiety, **(E)** unsafety, **(F)** loneliness.

Spatial visualization of emotional responses in Liwan District (Figure 4) revealed that positive emotions (pleasure, relaxation) were more prevalent in areas with high-quality urban features, such as public service zones, well-maintained alleyways, riverside green spaces, modern residential districts, and renovated urban villages. In contrast, lower-quality environments (e.g., industrial areas, underdeveloped spaces) exhibited heightened anxiety and unsafety. Spatial patterns inform policy priorities for green infrastructure-driven emotional wellbeing enhancement.

Pleasure and relaxation demonstrated spatial overlap, while curiosity and loneliness were diffusely distributed. Pleasure and relaxation had similar spatial distributions, with the highest intensity in public service areas (such as parks and educational institutions), riverside green belts, and modernized urban spaces (e.g., eastern Chajiao Street and Huadi Street). Key cultural and tourism hubs, like the Shamian Tourism Zone, Enning Road, and Zhongshan 8th Road, also elicited strong positive emotional responses, highlighting the significance of aesthetically and functionally optimized urban spaces. Major traffic corridors, including Huadi Avenue, Huangsha Avenue, and Longxi Avenue, contributed positively, probably because of their role in urban connectivity and development. In contrast, curiosity and loneliness had a more even spatial distribution, with fewer distinct high–perception areas.

Anxiety and unsafety clustered in lower-quality environments, particularly in industrial zones (e.g., Longxi Panlong) and urban villages (e.g., Tanwei Village, Xijiao Village), where poor spatial quality, inadequate infrastructure, and limited greenery suppressed positive emotions. The Guangfo Sci-tech Innovation Cluster and western Datan Sha Island demonstrated similar trends, highlighting the negative impact of underdeveloped spaces. While loneliness was less pronounced district-wide, it was slightly elevated in southwestern Pearl River regions, likely due to lower population density and reduced urban vibrancy.

### 3.4 Relationship between street spatial features and emotional response

**Figure 5:**
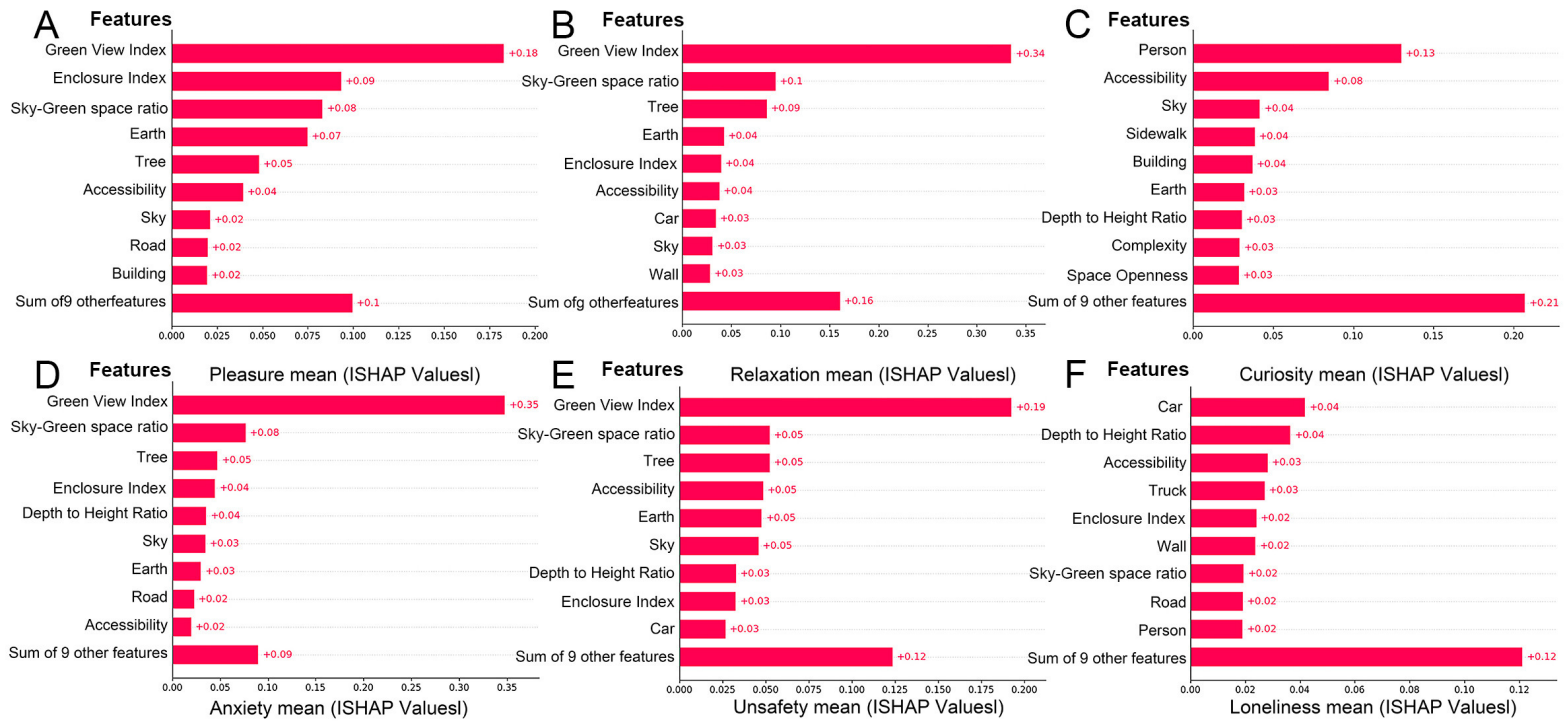
Feature importance of street environmental variables in predicting emotional responses (SHAP ranking). **(A)** Pleasure, **(B)** relaxation, **(C)** curiosity, **(D)** anxiety, **(E)** unsafety, **(F)** loneliness.

In the global sample, factors such as GVI, EI, sky-green space ratio, earth, trees, and accessibility significantly influence emotional responses and are key elements in street renovation. In contrast, elements such as walls, degree of motorization, cars, and trucks have a lower influence on emotional response (Figure 5). Walls, serving as static boundaries, lack the potential to evoke significant emotional fluctuations. Similarly, cars primarily serve as modes of transportation, and pedestrians tend to prioritize safety and mobility over the emotional impact of cars. However, under specific circumstances, such as artistic graffiti on walls or traffic congestion due to car accidents, these factors can become significant variables influencing emotional response.

#### 3.4.1 Influence of features on positive emotions

**Figure 6:**
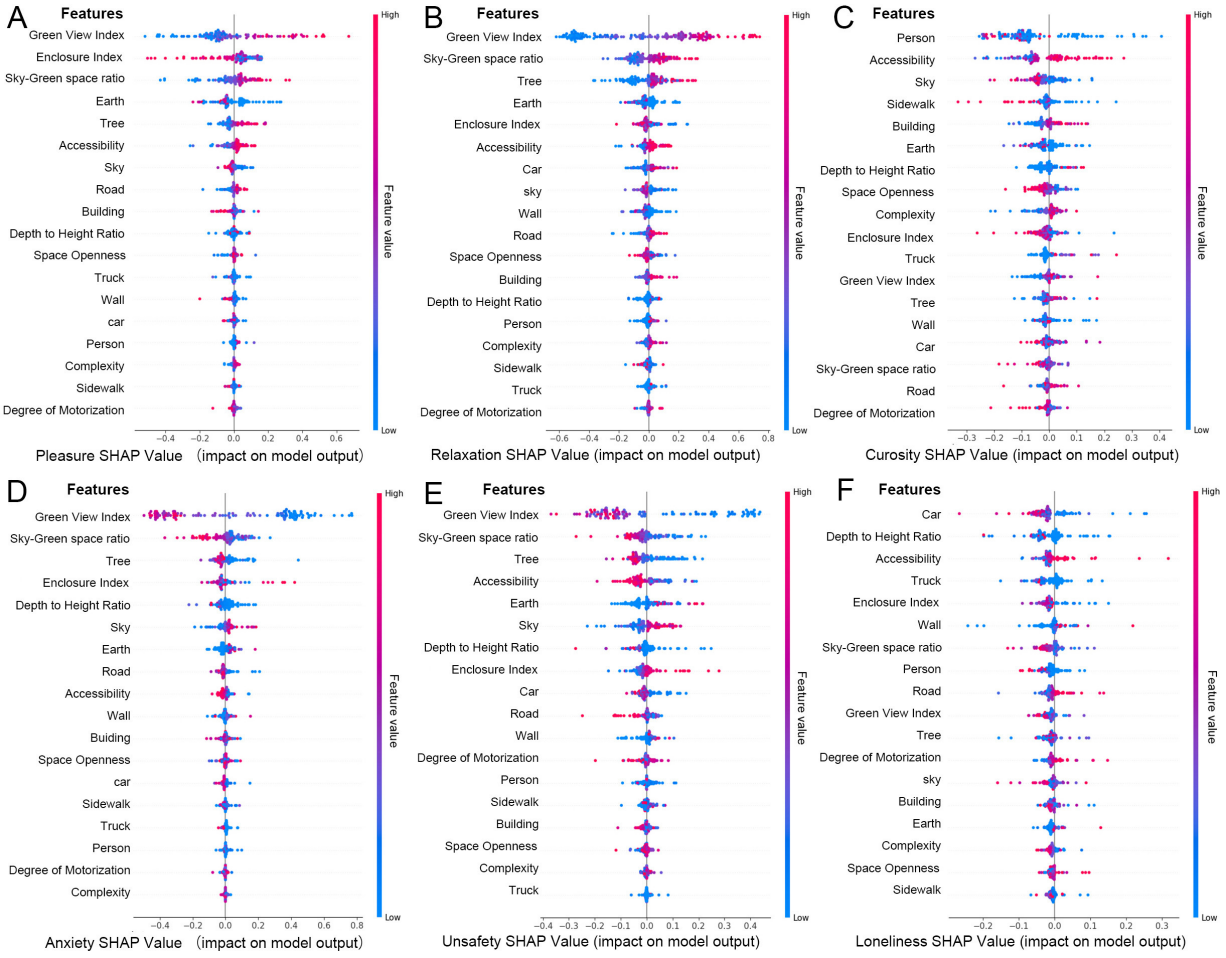
SHAP beeswarm plots of street features for predicting emotional responses. **(A)** Pleasure, **(B)** relaxation, **(C)** curiosity, **(D)** anxiety, **(E)** unsafety, **(F)** loneliness.

Figures 6A–C presents the SHAP beeswarm plots, which visualize the impact of each street spatial feature on model predictions of positive emotional responses, namely pleasure, relaxation, and curiosity. In these plots, each point represents a SHAP value for a single sample, with the color indicating the corresponding feature value (red: positive gain, blue: negative gain). Features are ordered by their overall importance, and the distribution of SHAP values illustrates both the magnitude and direction of each feature's effect.

The results indicate that pleasure and relaxation are strongly influenced by environmental features such as GVI, EI, sky-green space ratio, earth, and trees. Among them, GVI emerges as the most influential predictor of positive emotions, which aligns with previous research findings (56, 57). According to ART, natural environments capture involuntary attention, allowing individuals to disengage from intense cognitive activities and restore attentional resources (58). Stress Reduction Theory (SRT) further posits that elements in natural environments, such as green plants and water bodies, can reduce both physiological and psychological stress levels (59).

In particular, a moderate GVI (e.g., 20%−40%) not only increases time spent in the street but also significantly reduces anxiety. However, overly homogeneous or functionally inadequate green spaces may reduce these positive effects (60). EI reflects the integrated effect of buildings and natural features in shaping an enclosed space; when paired with ample vegetation and complete facilities, high enclosure can enhance feelings of pleasure, while narrow or dimly lit spaces may impair perceptual comfort. Similarly, the sky-green space ratio promotes both pleasure and relaxation, with visual openness and perceived comfort playing key roles.

The earth is found to be negatively correlated with pleasure when poorly maintained but enhances walking experience when well-paved. The sky, as an element contributing to openness, is positively associated with pleasure. Trees, a major component of GVI, further amplify the psychological benefits of street greenery by adding depth and vertical layering to the visual field.

On the other hand, curiosity, as a more dynamic and individualized emotion, exhibits distinct influencing factors. As shown in Figure 6C, crowd density, accessibility, sky, and sidewalk design are key drivers of curiosity. Unlike pleasure or relaxation, which are primarily evoked by natural features, curiosity is more closely tied to the narrative quality and spatial complexity of the urban environment. People with different spatial preferences may be drawn to either tranquil, green settings or culturally complex and functionally diverse spaces. In particular, built environments with historical significance or unique design features appear to stimulate stronger exploratory impulses.

#### 3.4.2 Influence of features on positive emotions

The mechanisms behind negative emotions are more complex. Anxiety and unsafety are closely related, primarily influenced by the GVI, sky-green space ratio, and trees (Figures 6D–F). These factors affect negative emotions in a manner opposite to positive emotions. A higher GVI alleviates anxiety and unsafety, while a lower index is linked to disordered environments. Traffic flow, especially congestion, increases noise and reduces efficiency, contributing to anxiety and unsafety.

Additionally, a moderate sky view, such as the sense of openness in rural or suburban areas, can also help alleviate anxiety. Furthermore, vibrant commercial areas and high-grade public service facilities, which foster active crowds and well-equipped environments, positively contribute to reducing anxiety.

The sky-green space ratio complements the GVI by compensating for the lack of sky visibility. Lower sky-green space ratio tends to be accompanied by stronger feelings of anxiety and unsafety. The role of vegetation is consistent with the GVI, but its impact is more concentrated. To mitigate these negative feelings, optimizing pedestrian infrastructure and increasing commercial activity in underutilized areas can significantly enhance street vitality and create a safe and comfortable atmosphere. Busy commercial areas can promote pedestrian activity, increase natural surveillance, and foster a sense of community belonging, thereby reducing feelings of anxiety and unsafety.

Following these factors, EI and accessibility also have significant effects on negative emotions. Anxiety may be exacerbated under high containment conditions, such as narrow lanes, obstructed views, or dimly lit alleys, while low traffic accessibility usually implies traffic congestion, which predisposes to feelings of unsafety. To alleviate these problems, strategies such as widening streets, improving pedestrian facilities, and improving lighting in enclosed areas may reduce the psychological impact of spatial enclosure and traffic congestion.

The overall incidence of loneliness is relatively low, but it is closely related to scene vitality and foot traffic density. Environments with sparse foot traffic are more likely to trigger feelings of isolation and loneliness. However, there were significant differences in individual responses to loneliness: familiar scenes or group activities were effective in alleviating loneliness, whereas desolate or activity-poor spaces may exacerbate the experience of loneliness.

#### 3.4.3 Spatial features and mixed emotional responses

The diversity of spatial features can trigger multiple emotional responses, reflecting significant interactional complexity. High GVI usually enhances pleasure and relaxation, but may induce loneliness under certain loneliness, depending on the spatial function and individual psychological state. The proportion of sky-green space ratio has a clear positive effect on positive emotions, and its visual and psychological functions play multiple roles in alleviating anxiety and stimulating curiosity; its ecological benefits (e.g., air purification, carbon neutrality) indirectly contribute to the overall enhancement of environmental quality and residents' emotional health. However, monotonous or poorly personalized designs can exacerbate feelings of loneliness.

EI exhibits a dual effect: a high degree of enclosure combined with rich natural elements and good facilities can stimulate positive emotions such as curiosity and relaxation, but insufficient lighting or oppressive spatial designs can exacerbate anxiety and unsafety. This duality is particularly evident in historic districts and commercial spaces, where the spatial features can simultaneously stimulate a sense of exploration while potentially causing stress due to enclosure or crowding.

Mixed emotional responses are nonlinear and context-dependent. By optimizing spatial design and harmonizing natural and artificial elements, urban spaces can better meet diverse emotional needs, enhancing emotional quality and providing higher emotional value, while improving ecological benefits.

### 3.5 Clustered coupling analysis of emotional response features

**Figure 7:**
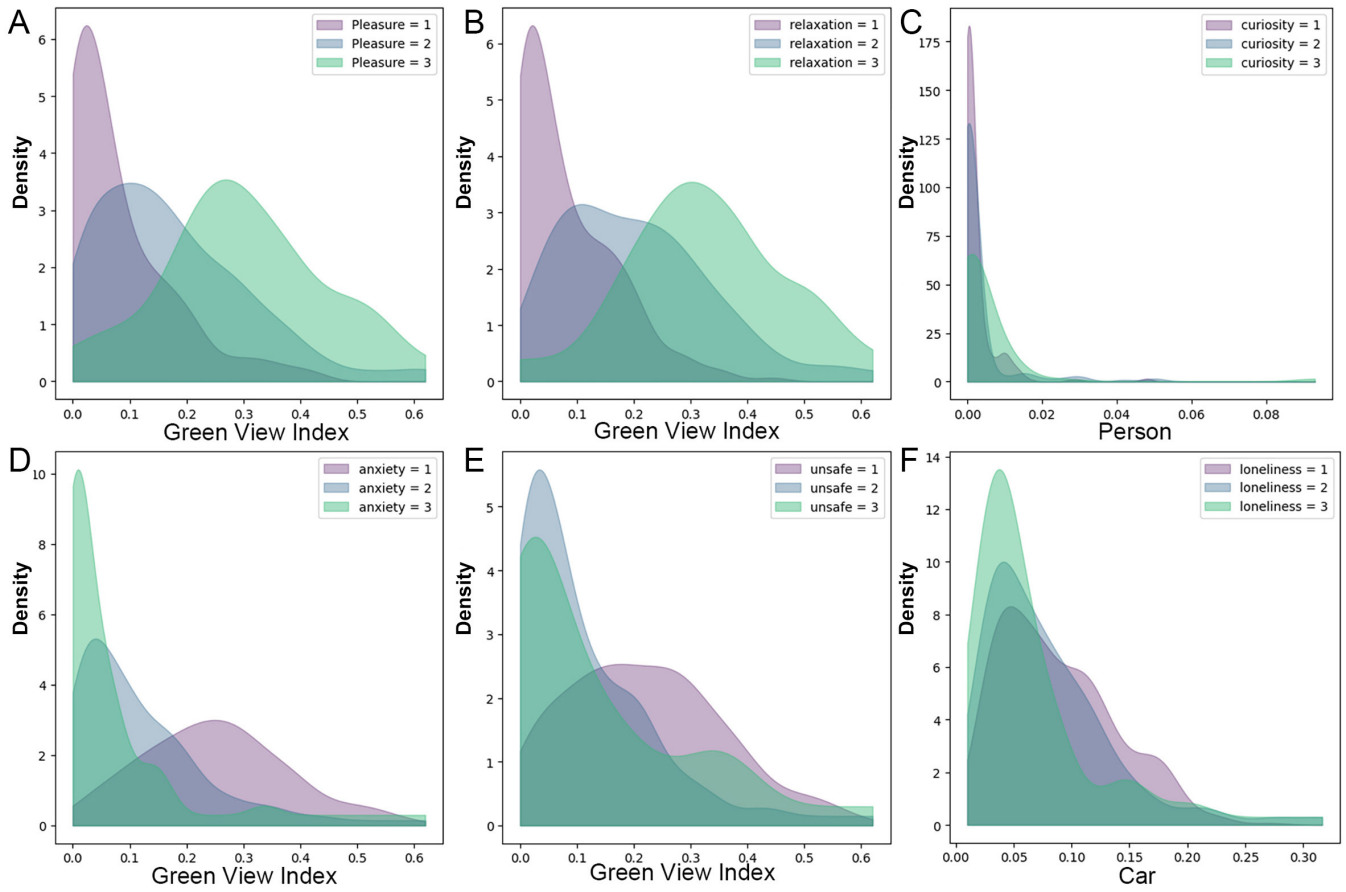
Heatmap of SHAP-derived interaction patterns between street features and emotional responses. **(A)** Pleasure, **(B)** relaxation, **(C)** curiosity, **(D)** anxiety, **(E)** unsafety, **(F)** loneliness.

Figure 7 shows the SHAP decision heatmap, with the *x*-axis representing 120 emotional response data samples and the color encoding representing the SHAP values of each factor. The baseline *f* (*x*) represents the model's predicted average value, where values above the baseline indicate high perception and those below indicates low perception. To clarify the interaction of multiple factors, the study uses SHAP results to segment the diagram with dashed boxes, highlighting the coupling mechanisms of primary factors influencing emotional response. This approach deepens the understanding of the relationship between the street environment and pedestrian emotional response, providing a foundation for urban street planning and design.

#### 3.5.1 Coupling patterns of positive emotions

For positive emotional responses, factors such as the GVI play significant regulatory roles: For pleasure, the synergy between the GVI, sky-green space ratio, and earth features, highlighted by the purple box, leads to a high pleasure perception. However, when these factors are coupled with EI, as shown in the rose box, a low pleasure perception emerges. For relaxation, the rose box is primarily influenced by the GVI, with blue-green space and tree coverage as key contributors, resulting in high relaxation perception. In contrast, areas where multiple factors combine tend to have a lower relaxation perception. As for curiosity, pedestrian presence stands out as the main driver. The purple and orange boxes highlight areas where pedestrian presence plays a major role, while other regions show a mix of pedestrian presence and additional factors.

#### 3.5.2 Coupling patterns of negative emotions

For negative emotional responses, the following patterns are observed: For anxiety, the green box shows high anxiety perception, influenced by the positive SHAP values of the GVI and EI, while the purple box shows low anxiety due to the negative impact of the GVI. Regarding unsafety, the purple box shows low perception, driven by the negative impact of the GVI, whereas the green box represents high unsafety perception, driven by its positive influence. The orange box lacks a dominant factor, resulting in low unsafety perception. In contrast, the rose box, although influenced positively by the GVI, is affected by other negative factors, leading to high unsafety perception. For loneliness, the green box reveals high loneliness perception, influenced by the positive effects of automobiles and the DH ratio. However, the negative coupling effect of traffic smoothness moderates this, resulting in an overall high loneliness perception. Other indicators show minor SHAP value differences, resulting in a lower loneliness perception through their coupled effects.

#### 3.5.3 Clustered features of mixed emotions

Overall, emotional responses are influenced by the complex coupling of multiple environmental features. The GVI is the most critical spatial factor, highlighting the importance of greenery and nature in promoting positive emotions. Meanwhile, sky-green space ratio, EI, and Accessibility serve as corrective indicators for emotional response results.

Factors such as earth, sky, buildings, and automobiles, while highly correlated with these composite indicators, exhibit weaker predictive power as single indicators. These factors can be further analyzed as supplementary components to refine the prediction results.

### 3.6 Analysis of the relationship between key spatial environmental features and emotional responses

By integrating Kernel Density Estimation (KDE) and SHAP value analysis (SHAP), this study systematically examines the impact mechanisms of key spatial environmental features, such as the GVI, tree ratio, and the sky-green space ratio, on emotional responses. The two methods complement each other in terms of spatial scale and explanatory depth. While KDE uncovers macro-level distribution patterns, SHAP delves into the nonlinear mechanisms at play. This integrated approach ultimately proposes a collaborative optimization strategy, offering a scientific foundation for high-density urban environment design.

#### 3.6.1 Kernel density estimation (KDE) analysis

**Figure 8:**
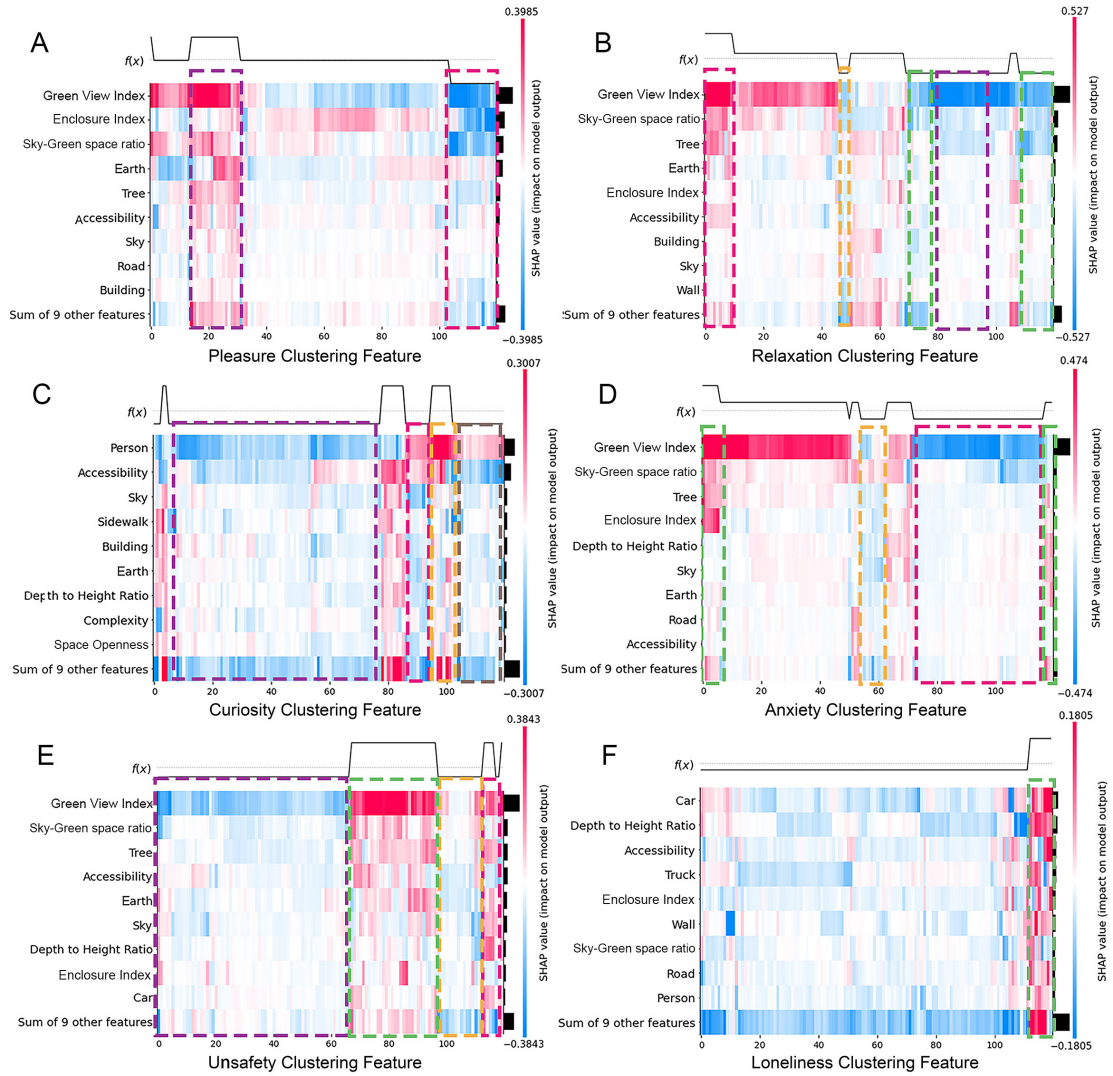
Kernel density distributions of environmental features by emotional response level. **(A)** Pleasure, **(B)** relaxation, **(C)** curiosity, **(D)** anxiety, **(E)** unsafety, **(F)** loneliness.

Using KDE, this study investigates the relationship between emotions and their most significant spatial environmental features (Figure 8). The results indicate significant differences in the distribution of feature factors across various emotional states. When the GVI is low (below 0.09), feelings of pleasantness and relaxation are concentrated in the lower levels. As the GVI increases, higher perceptions of pleasantness and relaxation gradually emerge, peaking at GVI values around 0.27 and 0.3. After this point, the increase is minimal, suggesting that the emotional improvement effect is primarily concentrated within this range. The optimal GVI threshold (0.27–0.3) provides clear guidance for urban renewal strategies.

Anxiety and unsafety show high densities at low GVI values (below 0.1). As the GVI increases, high-level perceptions decrease, while middle and low-level perceptions rise, indicating that higher GVI values effectively alleviate negative emotions. Low curiosity is notably higher in areas with pedestrian densities below 0.01, whereas high curiosity gradually prevails in areas with pedestrian densities above 0.02. High loneliness is most pronounced at vehicle densities below 0.05. Between 0.05 and 0.1, moderate loneliness peaks, while low loneliness gradually becomes dominant as vehicle density increases. The KDE method effectively highlights the influence of GVI on emotional responses at a spatial level, but it does not thoroughly explore the interactions and nonlinear relationships between features. This gap is addressed through SHAP feature dependence plots.

#### 3.6.2 SHAP analysis: nonlinear mechanism analysis

**Figure 9:**
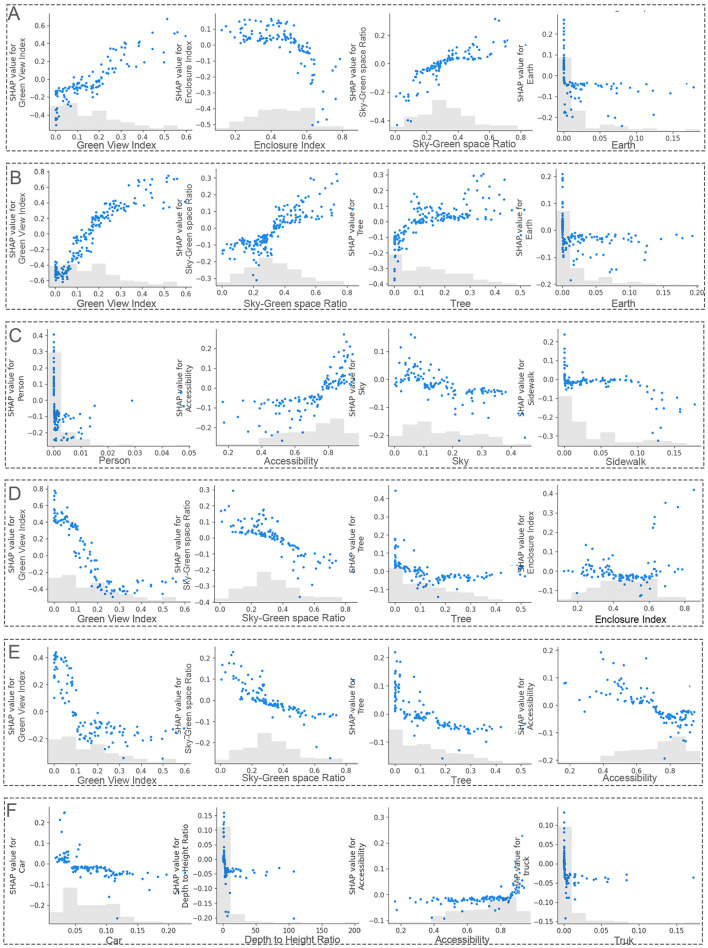
SHAP dependence plots illustrating nonlinear effects of environmental features on emotions. **(A)** Pleasure, **(B)** relaxation, **(C)** curiosity, **(D)** anxiety, **(E)** unsafety, **(F)** loneliness.

This study utilizes SHAP feature dependence plots to examine the nonlinear impact of environmental features on emotional responses (Figure 9). By analyzing the SHAP plots of six urban environmental features with significant emotional impacts, we gain a deeper understanding of their contributions to emotional responses.

An increase in GVI enhances pleasantness and relaxation while reducing anxiety and unsafety. Below a GVI of 0.3, the improvements in pleasantness and relaxation are most noticeable, especially for relaxation. As the GVI exceeds 0.3, the effect on emotional improvement weakens, and after surpassing 0.35, further emotional benefits are minimal. This finding aligns with the KDE analysis, confirming the optimal range of GVI between 0.27 and 0.3. Notably, when GVI exceeds 0.5, pleasantness decreases slightly, and unsafety and anxiety increase. Thus, maintaining a GVI below 0.35 avoids the negative effects of overly high GVI.

Tree cover percentage shows significant nonlinear effects as well. In the 0–0.1 range, increasing tree cover enhances pleasantness and relaxation, with the effect strengthening as tree cover increases. However, beyond 0.35, the canopy shading effect creates a sense of spatial confinement, weakening emotional benefits. Therefore, tree ratio should be maintained below 0.35 to avoid discomfort caused by excessive tree cover.

Regarding the sky-green space ratio, the physical structure of buildings limits spatial layout, making it difficult to improve this ratio solely by adjusting building coverage. Optimizing GVI, however, can indirectly enhance it and thus serves as an effective strategy. In high-density urban areas, improving GVI not only increases the sky-green space ratio but also alleviates negative emotions caused by high building density, significantly improving emotional health.

## 4 Discussion

This study utilizes big data and machine learning techniques to predict emotional responses based on street view images, revealing the mechanisms by which urban spatial environments influence emotions and their intrinsic connection to public health. By constructing urban emotional hotspot maps, the study provides a scientific basis for identifying emotionally vulnerable areas, particularly in relation to key public health issues such as disparities in green space and mental health (4, 61, 62). Using Liwan District, Guangzhou as a case study, it analyzes the spatial heterogeneity of emotional responses and the critical role of street spatial visual features, offering empirical evidence and references for future public health research on urban green space interventions.

### 4.1 Multidimensional emotional benefits and spatial optimization mechanisms of street environment features

The integration of KDE and SHAP establishes a collaborative approach that quantifies spatial heterogeneity and decodes nonlinear relationships. KDE, through spatial clustering analysis, identifies the critical improvement threshold for the GVI (0.27–0.30). SHAP reveals complex nonlinear dynamics by quantifying the diminishing marginal effects of GVI beyond 0.35, identifying the synergistic interaction between the sky-green space ratio and tree cover (63, 64), and uncovers threshold-driven emotional response patterns (e.g., a sharp drop in certain emotions when GVI < 0.1). Notably, our identification of an optimal GVI range echoes the concept of defining sufficient green exposure in high-density cities (65), providing quantitative benchmarks for greening interventions that maximize emotional benefits. This framework bridges the gap between spatial goals and causal explanations, demonstrating that optimizing GVI, tree cover, and blue-green spaces requires balancing threshold effects and feature interactions to achieve a dynamic equilibrium between environmental features and emotional wellbeing.

Empirical examples, such as Singapore's “Sky Garden” program (66), show how vertical greening and integrated green spaces enhance emotional health, air quality, and urban resilience. In high-density urban areas, cost-effective interventions—such as vertical greening systems, strategically positioned community green spaces (e.g., pocket parks), and street micro-upgrading strategies—are prioritized to increase the baseline GVI from < 0.1 to ~0.27–0.30 while minimizing spatial conflicts in resource-constrained contexts (67, 68). However, as demonstrated in Table 6, even streets with high GVI may fail to evoke positive emotional perceptions if they are accompanied by poor tree pruning, monotonous environments, frequent illegal parking, insufficient maintenance, or homogeneous greening layouts. These findings highlight the importance of combining quantitative GVI targets with qualitative enhancements, such as diverse vegetation layouts, art installations, and cultural features, to expand spatial attractiveness and emotional benefits.

**Table 6:** Characteristics of streets with a high GVI.

**Visual commonality**	**Perceptual commonality**	**Reference images and their characteristics**
High GVI	High pleasure	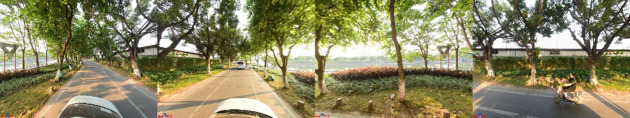 The street greening layout adopts a “two-board-three-belt” or “one-board-two-belt” structure, offering shading that ensures both visual and physiological comfort. This design improves driving safety and landscape continuity. Trees are pruned to maintain clear sightlines, while aligned forest edges and coordinated plant species and layers emphasize spatial order and aesthetic value.
High GVI	Medium pleasure	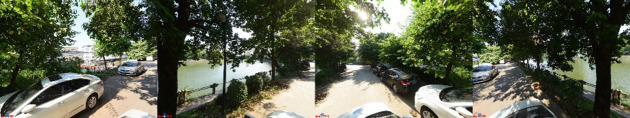 Though the GVR meets the standard, inadequate pruning of street trees creates a monotonous atmosphere. On-street parking often disrupts the street's order. Additionally, poor rural road greening maintenance results in a lack of environmental harmony.
High GVI	High relaxation	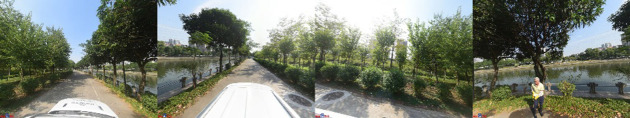 The greening structure is varied, with rich landscape elements. Roadside green belts emphasize plant diversity while integrating landscape and ecological functions. These spaces also include leisure characteristics, balancing aesthetics and functionality.
High GVI	Medium relaxation	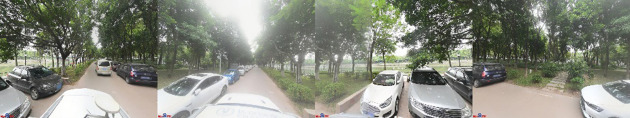 The street greening structure is monotonous, lacking layers and spatial design. The layout is highly repetitive and lacks variability, hindering the integration of diverse landscape elements. Additionally, the focus on vegetation ignores functional and aesthetic values. Rampant on-street parking worsens spatial disorder, degrading the overall landscape quality.

Layered vegetation design further meets functional needs: residential areas benefit from tree cover between 25 and 35% to mitigate spatial monotony, while commercial areas may reduce it to 10%−20% to alleviate visual congestion. EI should be evaluated in the context of emotional perception. Moderate enclosures enhance perceived safety and comfort (69, 70), whereas excessive enclosures create a sense of oppression, and overly open layouts decrease spatial legibility and emotional attachment (71, 72). The level of enclosure should be adjusted to strengthen place identity, regulate pedestrian flow, and maintain visual connectivity—critical considerations for designing urban environments informed by mental health.

Implementation depends on cross-sector collaboration (government-community-resident partnerships), supported by fiscal incentives (e.g., tax incentives and grants) (73–75), to ensure equitable green space distribution. Future efforts should integrate pedestrian-friendly design with cultural aesthetics to foster social cohesion (76), while further case studies are necessary to clarify the spatiotemporal heterogeneity of EI effects.

### 4.2 Data-driven urban emotional response optimization

The emotional maps derived from street view big data and predictive models provide detailed spatial insights for identifying emotional distress hotspots. This data-driven framework lays an empirical foundation for urban optimization, enabling decision-makers to deploy targeted interventions that enhance public health and environmental sustainability. Strategically integrating multifunctional infrastructure, such as pedestrian corridors, community green spaces, and cultural centers, in emotionally vulnerable areas can strengthen place attachment and urban health resilience (77). Prioritizing the deployment of green infrastructure in high-stress areas can improve emotional health while optimizing resource efficiency. Predictive models ensure that interventions align with the actual needs of the city and prevent environmentally harmful developments (78–80).

Advances in data analysis technologies allow urban planners to adopt more systematic, data-driven strategies. The digital governance projects of the Guangzhou government, such as the “Smart Tower Patrol” and the “Smart Governance Portal,” support the high-quality development of urban renewal and digital transformation, enhancing urban space quality (81, 82). By combining emotional maps with multidimensional environmental data such as temperature changes, noise levels, and greenery coverage, cities can implement real-time monitoring systems to track emotional changes within urban spaces. These monitoring mechanisms provide timely feedback on environmental changes, enabling cities to quickly respond to crises such as public health emergencies or extreme weather events. For instance, during public health emergencies, real-time emotional monitoring can guide targeted interventions, including prioritizing air quality improvements and increasing green space accessibility, thus reducing anxiety and enhancing public wellbeing (83, 84).

Furthermore, long-term dynamic data analysis plays a crucial role in assessing the lasting effects of environmental transformations. Continuous evaluation of urban renewal allows policymakers to refine and adjust strategies, ensuring sustainable and regenerative urban growth. This iterative approach strengthens evidence-based policymaking, enhancing the connection between emotional wellbeing and urban resilience, and promoting healthier, more adaptable urban environments.

### 4.3 Social equity in urban emotional optimization

Optimizing emotional responses in urban environments is closely aligned with the pursuit of social equity. Prior research shows that neighborhoods with lower socio-economic status often exhibit reduced streetscape quality, contributing to emotional vulnerability and spatial health disparities (4). Visual environmental inequities—such as limited greenery or visual clutter—intensify negative emotions like anxiety and insecurity. Targeted environmental improvements in such areas have been shown to enhance emotional wellbeing and strengthen public health resilience (85).

Extending this evidence, our study reveals a significant association between low environmental quality—particularly diminished GVI—and clusters of negative emotional responses in disadvantaged communities. These findings emphasize the importance of addressing visual inequities to strengthen neighborhood emotional resilience and reduce disparities in both mental health and environmental access. This aligns with the social equity principles embedded in the United Nations Sustainable Development Goals (SDGs). For example, prioritizing green infrastructure in under-served neighborhoods ensures that the benefits of sustainable urban development are equitably distributed (86).

However, due to inequitable resource allocation, spatially disadvantaged communities often face financial and technical barriers. To overcome these challenges, three strategies have been proposed:

1. Perceptually driven prioritization: Urban policy should prioritize environmental improvements in areas where spatial features are strongly linked to negative emotional responses such as anxiety, unsafety, and disconnection. These areas, often characterized by fragmented form, low greenery, and poor walkability, are critical for emotional restoration. Rather than relying solely on greening indices, interventions should incorporate adaptive strategies—such as enhancing spatial legibility, improving pedestrian comfort, and enriching visual diversity—to address perceptual deficits. This emotion-informed allocation ensures that public investment aligns with psychological needs and promotes equity in urban health outcomes.
2. Participatory neighborhood Co-creation: Empowering residents to participate in neighborhood design fosters a sense of ownership and emotional attachment. Collaborative mechanisms, including financial incentives and partnerships with local businesses, can increase participation and social cohesion. The “Community Garden Program” in the United States illustrates how collective action can enhance both environmental quality and community wellbeing.
3. Sustained institutional support: Government subsidies and incentive policies play a crucial role in sustaining green infrastructure. Our findings underscore that the psychological gains from environmental improvements—such as reductions in anxiety and stress—are contingent on proper maintenance; without continuous support, these emotional benefits will likely diminish over time. By reducing long-term maintenance costs associated with neighborhood optimization, these mechanisms promote the durability of urban greening efforts. Additionally, public-private partnership (PPP) models provide financial support for maintaining and expanding green infrastructure, ensuring long-term viability and reinforcing environmental sustainability.

Implementing these strategies enhances urban environments while fostering social equity, ensuring all community members benefit from sustainable development and equitable access to green spaces.

### 4.4 Future research and technological advancements

Optimizing emotional responses in urban environments necessitates a convergence of interdisciplinary insights. Psychological and ecological studies have illuminated the mechanisms by which natural environments mitigate stress and restore attention, while sociological research emphasizes the role of green infrastructure in enhancing community cohesion and perceived safety. Additionally, economic evaluations provide critical support for cost-effective decision-making in health-oriented urban planning.

Building on this foundation, the present study contributes to the field across three interrelated dimensions—methodological innovation, empirical validation, and planning applicability—thereby addressing key limitations in existing research and informing future urban interventions. First, this study establishes a novel analytical framework that integrates deep learning, explainable AI (SHAP), and geospatial analysis to uncover the complex and nonlinear relationships between street-level visual features and emotional responses. Compared with traditional linear models, this approach enhances interpretability and accommodates the subjective nature of emotional perception, addressing the oversimplifications found in prior studies (13, 50).

Second, through large-scale empirical analysis, the study quantifies the emotional effects of specific spatial features, particularly Green View Index (GVI) and the sky-green space ratio. It identifies optimal thresholds (e.g., GVI between 0.27–0.30) and diminishing marginal effects beyond certain values, revealing nonlinear dynamics that extend existing literature on urban greening and mental health (17).

Third, the findings are translated into practical urban design guidance, emphasizing evidence-based and context-sensitive interventions. These include vertical greening in high-density settings, pocket parks in constrained spaces, and improvements to visual diversity, all of which are aimed at enhancing emotional wellbeing while ensuring spatial feasibility and sustainability.

Despite these advancements, several technological and methodological challenges persist, warranting future research. A primary obstacle lies in data acquisition. Current street-view imagery systems—typically reliant on vehicle-mounted panoramic cameras—fail to capture fine-grained spatial experiences in narrow alleys, shaded corridors, or enclosed public spaces. Future studies should consider integrating drone-based imagery to access elevated and confined views (77), and pedestrian-worn sensors, such as EEG and biometric trackers, to capture real-time emotional fluctuations in response to specific micro-environmental stimuli.

In parallel, further development is needed in emotionally aware analytical models. While current deep learning models demonstrate robust performance in object detection and classification, they struggle with culturally diverse or morphologically complex urban environments. The integration of context-sensitive algorithms—capable of incorporating architectural typologies, cultural markers, and localized spatial semantics—may substantially improve the accuracy and generalizability of emotional prediction models (87). Such models should move beyond purely visual cues to consider multimodal inputs, including acoustic environments and air quality indicators.

Moreover, the advancement of real-time emotional monitoring systems offers a compelling direction for urban governance. By embedding IoT-enabled environmental sensors into public infrastructure and coupling them with dynamic emotional maps, planners can monitor emotional patterns across temporal and spatial scales. These systems enable cities to respond adaptively to evolving urban stressors such as extreme weather, pollution surges, or public health emergencies (88). Feedback loops informed by real-time data could support flexible policy adjustments, emergency interventions, and longitudinal evaluation of environmental upgrades.

Taken together, these future directions underscore the necessity of combining methodological rigor, technological innovation, and socio-spatial sensitivity to develop emotionally intelligent cities. A truly health-promoting urban environment must not only be efficient and sustainable but also responsive to the affective experiences of its inhabitants.

## 5 Conclusions

This study integrates deep learning, explainable AI, and geospatial analysis to quantify how spatial environmental features influence emotional responses. By leveraging large-scale street-view imagery and machine learning models, it establishes a robust framework for identifying key visual factors—such as GVI, sky-green space ratio, and EI—that exhibit significant and nonlinear effects on emotional perception. These findings reveal spatially clustered patterns of emotional vulnerability, offering a scientific basis for targeted interventions that support psychological resilience and public health.

Aligned with the United Nations Sustainable Development Goals, particularly SDG 3 (Good Health and wellbeing) and SDG 11 (Sustainable Cities and Communities), the study highlights the potential of data-informed spatial optimization to enhance emotional wellbeing and urban sustainability.

Future research may further explore the temporal variability and socio-cultural dimensions of emotional responses, while emerging technologies—such as IoT and dynamic emotional mapping—could improve the precision and adaptability of future urban planning strategies.

## Data Availability

The original contributions presented in the study are included in the article/supplementary material, further inquiries can be directed to the corresponding author.

## References

[1] Gehl J. Cities for People. Washington, DC: Island Press (2013).

[2] Gianfredi V Buffoli M Rebecchi A Croci R Oradini-Alacreu A Stirparo G. Association between urban greenspace and health: a systematic review of literature. Int J Environ Res Public Health. (2021) 18:5137. 10.3390/ijerph18105137 34066187 PMC8150317

[3] Gascon M Triguero-Mas M Martínez D Dadvand P Forns J Plasència A. Mental health benefits of long-term exposure to residential green and blue spaces: a systematic review. Int J Environ Res Public Health. (2015) 12:4354–79. 10.3390/ijerph120404354 25913182 PMC4410252

[4] Wang S Yoo J Cai W Yang F Huang X Sun QC. Reducing the social inequity of neighborhood visual environment in Los Angeles through computer vision and multi-model machine learning. Sustain Cities Soc. (2025) 119:106062. 10.1016/j.scs.2024.106062 39896741 PMC11781154

[5] Favarão Leão AL Banda B Xing E Gudapati S Ahmad A Lin J. Applications of artificial intelligence in public health: analyzing the built environment and addressing spatial inequities. J Public Health. (2025) 1–11 10.1007/s10389-025-02444-x

[6] Degen MM Rose G. The sensory experiencing of urban design: the role of walking and perceptual memory. Urban Stud. (2012) 49:3271–87. 10.1177/0042098012440463

[7] Abusaada H. Strengthening the affectivity of atmospheres in urban environments: the toolkit of multi-sensory experience. ArchNet-IJAR. (2020) 14:379–92. 10.1108/ARCH-03-2020-0039

[8] Hollander JB Anderson EC. The impact of urban façade quality on affective feelings. ArchNet-IJAR. (2020) 14:219–32. 10.1108/ARCH-07-2019-0181

[9] Barrett LF Russell AA. The structure of current affect: controversies and emerging consensus. Curr Dir Psychol Sci. (1999) 8:10–4. 10.1111/1467-8721.00003

[10] Banaei M Ahmadi A Gramann K Hatami J. Emotional evaluation of architectural interior forms based on personality differences using virtual reality. Front Archit Res. (2020) 9:138–47. 10.1016/j.foar.2019.07.005

[11] Knobel P Maneja R Bartoll X Alonso L Bauwelinck M Valentin A. Quality of urban green spaces influences residents' use of these spaces, physical activity, and overweight/obesity. Environ Pollut. (2021) 271:116393. 10.1016/j.envpol.2020.116393 33388678

[12] Qiao YH Chen ZN Chen YQ Zheng TX. Deciphering the link between mental health and green space in Shenzhen, China: the mediating impact of residents' satisfaction. Front Public Health. (2021) 9:561809. 10.3389/fpubh.2021.561809 33643984 PMC7902702

[13] Zhang J Yu Z Li Y Wang X. Uncovering bias in objective mapping and subjective perception of urban building functionality: a machine learning approach to urban spatial perception. Land. (2023) 12:1322. 10.3390/land12071322

[14] Qin J Feng Y Sheng Y Huang Y Zhang F Zhang K. Evaluation of pedestrian-perceived comfort on urban streets using multi-source data: a case study in Nanjing, China. ISPRS Int J Geo-Inf. (2025) 14:63. 10.3390/ijgi14020063

[15] Weijs-Perrée M Dane G van den Berg P van Dorst M. A multi-level path analysis of the relationships between the momentary experience characteristics, satisfaction with urban public spaces, and momentary- and long-term subjective wellbeing. Int J Environ Res Public Health. (2019) 16:3621. 10.3390/ijerph16193621 31561634 PMC6801588

[16] Peng H Zhu T Yang T Zeng M Tan S Yan L. Depression or recovery? A study of the influencing elements of urban street environments to alleviate mental stress. Front Archit Res. (2025) 14:846–62. 10.1016/j.foar.2024.11.006

[17] Hao N Li X Han D Nie W. Quantifying the impact of street greening during full-leaf seasons on emotional perception: guidelines for resident well-being. Forests. (2024) 15:119. 10.3390/f15010119

[18] Long Y Liu L. How green are the streets? An analysis for central areas of Chinese cities using Tencent Street view. PLoS ONE. (2017) 12:e0171110. 10.1371/journal.pone.0171110 28196071 PMC5308808

[19] Wu D Gong JH Liang JM Sun J Zhang GY. Analyzing the influence of urban street greening and street buildings on summertime air pollution based on street view image data. ISPRS Int J Geo-Inf. (2020) 9:500. 10.3390/ijgi9090500

[20] Yin L Wang ZX. Measuring visual enclosure for street walkability: using machine learning algorithms and google street view imagery. Appl Geogr. (2016) 76:147–53. 10.1016/j.apgeog.2016.09.024

[21] Yang CC Xu FN Jiang L Wang R Yin L Zhao M. Approach to quantify spatial comfort of urban roads based on street view images. J Geo-Inform Sci. (2021) 23:785–801. 10.12082/dqxxkx.2021.200353

[22] Row AT. The Death and Life of Great American Cities. New York, NY: Random House (1962).

[23] Gehl J. Life Between Buildings: Using Public Space. New York, NY (2011).

[24] Rundle AG Bader MDM Richards CA Neckerman KM Teitler JO. Using google street view to audit neighborhood environments. Am J Prev Med. (2011) 40:94–100. 10.1016/j.amepre.2010.09.034 21146773 PMC3031144

[25] Fang CY Homma R Qiu TF. A bibliometrics analysis related to the built environment and walking. Sustainability. (2024) 16:2850. 10.3390/su16072850

[26] Zhang SL Chen L. Acoustic information masking effects of natural sounds on traffic noise based on psychological health in open urban spaces. Front Public Health. (2023) 11:1031501. 10.3389/fpubh.2023.1031501 36935713 PMC10022823

[27] Chen CJ Cao Y Xu GF Zhong Q Chen B. How do urban block built environments affect older adults' walking activities and health effects: a case study in Nanjing, China. Front Public Health. (2024) 12:1479305. 10.3389/fpubh.2024.1479305 39403438 PMC11471666

[28] Lee JH Ostwald MJ. Mathematical beauty and Palladian architecture: measuring and comparing visual complexity and diversity. Front Archit Res. (2024) 13:729–40. 10.1016/j.foar.2024.03.004

[29] Lorenz WE Kulcke M. Multilayered complexity analysis in architectural design: two measurement methods evaluating self-similarity and complexity. Fractal fract. (2021) 5:244. 10.3390/fractalfract5040244

[30] Akgün-Tanbay N Campisi T Tanbay T Tesoriere G Dissanayake D. Modelling road user perceptions towards safety, comfort, and chaos at shared space: the via maqueda case study, Italy. J Adv Transp. (2022) 2022:1–3. 10.1155/2022/4979496

[31] Bivina GR Parida M. Prioritizing pedestrian needs using a multi-criteria decision approach for a sustainable built environment in the Indian context. Environ Dev Sustain. (2020) 22:4929–50. 10.1007/s10668-019-00381-w

[32] Xie HF Liu L Yue H. Modeling the effect of streetscape environment on crime using street view images and interpretable machine-learning technique. Int J Environ Res Public Health. (2022) 19:13833. 10.3390/ijerph192113833 36360717 PMC9655263

[33] Li S Walters G Packer J Scott N. Using skin conductance and facial electromyography to measure emotional responses to tourism advertising. Curr Issues Tour. (2018) 21:1761–83. 10.1080/13683500.2016.1223023

[34] Kaplan R Kaplan S. The Experience of Nature: A Psychological Perspective. Cambridge: Cambridge University Press (1989).

[35] Gath-Morad M Plaut PO Kalay YE. Attract or repel: how street features shape pedestrians' leisure walks in cities. J Urban Design. (2024) 29:342–62. 10.1080/13574809.2023.2237468

[36] Sones M Holden M Kestens Y King AC Rennie M Winters M. (Dis)connected by design? Using participatory citizen science to uncover environmental determinants of social connectedness for youth in under-resourced neighbourhoods. BMC Public Health. (2024) 24:3104. 10.1186/s12889-024-20597-4 39529084 PMC11552136

[37] He KM Gkioxari G Dollár P Girshick R. Mask R-CNN. IEEE Trans Pattern Anal Mach Intell. (2020) 42:386–97. 10.1109/TPAMI.2018.2844175 29994331

[38] Zhao HS Shi JP Qi XJ Wang XG Jia JY. Pyramid scene parsing network. In: 30th IEEE/CVF Conference on Computer Vision and Pattern Recognition (CVPR). Honolulu, HI: IEEE (2017). p. 6230–9. 10.1109/CVPR.2017.660

[39] Zhang F Zhou BL Liu L Liu Y Fung HH Lin H. Measuring human perceptions of a large-scale urban region using machine learning. Landsc Urban Plann. (2018) 180:148–60. 10.1016/j.landurbplan.2018.08.020

[40] Qi Y Drolma SC Zhang X Liang J Jiang HB Xu JG. An investigation of the visual features of urban street vitality using a convolutional neural network. Geo-Spat Inf Sci. (2020) 23:341–51. 10.1080/10095020.2020.1847002

[41] Liu W Li D Meng Y Guo CM. The relationship between emotional perception and high-density built environment based on social media data: evidence from spatial analyses in Wuhan. Land. (2024) 13:294. 10.3390/land13030294

[42] Asrat KT Cho HJ. A comprehensive survey on high-definition map generation and maintenance. ISPRS Int J Geo-Inf. (2024) 13:232. 10.3390/ijgi13070232

[43] Zhang F Salazar-Miranda A Duarte F Vale L Hack G Chen M. Urban visual intelligence: studying cities with artificial intelligence and street-level imagery. Ann Am Assoc Geogr. (2024) 114:876–97. 10.1080/24694452.2024.2313515

[44] Ren M Zhang XF Zhi XB Wei YJ Feng ZY. An annotated street view image dataset for automated road damage detection. Sci Data. (2024) 11:407. 10.1038/s41597-024-03263-7 38649712 PMC11035563

[45] Colombo M Pincay J Lavrovsky O Iseli L Van Wezemael J Portmann E. Streetwise: mapping citizens' perceived spatial qualities. In: Proceedings of the 23rd International Conference on Enterprise Information Systems (ICEIS 2021), Vol 1. Lisbon: SciTePress (2021). p. 810–8. 10.5220/0010532208100818

[46] Helbich M Yao Y Liu Y Zhang JB Liu PH Wang RY. Using deep learning to examine street view green and blue spaces and their associations with geriatric depression in Beijing, China. Environ Int. (2019) 126:107–17. 10.1016/j.envint.2019.02.013 30797100 PMC6437315

[47] Wang L Han X He J Jung T. Measuring residents' perceptions of city streets to inform better street planning through deep learning and space syntax. ISPRS J Photogramm Remote Sens. (2022) 190:215–30. 10.1016/j.isprsjprs.2022.06.011

[48] Wang RY Cao MQ Yao Y Wu WJ. The inequalities of different dimensions of visible street urban green space provision: a machine learning approach. Land Use Policy. (2022) 123:106410. 10.1016/j.landusepol.2022.106410

[49] Yue YF Yang DF Van Dyck D. Urban greenspace and mental health in Chinese older adults: associations across different greenspace measures and mediating effects of environmental perceptions. Health Place. (2022) 76:102856. 10.1016/j.healthplace.2022.102856 35803043

[50] Lu X Li Q Ji X Sun D Meng Y Yu Y. Impact of streetscape built environment characteristics on human perceptions using street view imagery and deep learning: a case study of Changbai Island, Shenyang. Buildings. (2025) 15:1524. 10.3390/buildings15091524

[51] Ouyang YF Bai XH Wang XC Chen YL Huang GS Xie DX. Case study on cultural industry empowerment in urban renewal: a focus on Guangzhou, China. Sustainability. (2025) 17:439. 10.3390/su17020439

[52] Ma Y Brindley P Lange E. From modelling and analysis of accessibility of urban green space to green infrastructure planning: Guangzhou as a case study. In: Adaptive Urban Transformation: Urban Landscape Dynamics, Regional Design and Territorial Governance in the Pearl River Delta, China. Cham: Springer International Publishing (2023). p. 249–66. 10.1007/978-3-030-89828-1_13

[53] Breiman L. Random forests. Mach Learn. (2001) 45:5–32. 10.1023/A:1010950718922

[54] Luo YY Liu YF Tong ZM Wang NN Rao L. Capturing gender-age thresholds disparities in built environment factors affecting injurious traffic crashes. Travel Behav Soc. (2023) 30:21–37. 10.1016/j.tbs.2022.08.003

[55] Lundberg SM Lee SI. A unified approach to interpreting model predictions. In: 31st Annual Conference on Neural Information Processing Systems (NIPS), Vol 30. Long Beach, CA (2017).

[56] Nguyen PY Astell-Burt T Rahimi-Ardabili H Feng XQ. Green space quality and health: a systematic review. Int J Environ Res Public Health. (2021) 18:11028. 10.3390/ijerph182111028 34769549 PMC8582763

[57] Peschardt KK Stigsdotter UK Schipperrijn J. Identifying features of pocket parks that may be related to health promoting use. Landsc Res. (2016) 41:79–94. 10.1080/01426397.2014.894006

[58] Katz C. The experience of nature - a psychological perspective. J Nerv Ment Dis. (1991) 179:704. 10.1097/00005053-199111000-00012

[59] Ulrich RS. Stress reduction theory. In:Marchand D Pol E Weiss K, editors. 100 Key Concepts in Environmental Psychology. New York, NY: Routledge (2023). p. 143–6.

[60] Schreuder E van Erp J Toet A Kallen VL. Emotional responses to multisensory environmental stimuli: a conceptual framework and literature review. Sage Open. (2016) 6:1–19. 10.1177/2158244016630591

[61] Liang XC Chang JH Gao S Zhao TH Biljecki F. Evaluating human perception of building exteriors using street view imagery. Build Environ. (2024) 263:111875. 10.1016/j.buildenv.2024.111875

[62] Chen CX Li HW Luo WJ Xie JH Yao J Wu LF. Predicting the effect of street environment on residents' mood states in large urban areas using machine learning and street view images. Sci Total Environ. (2022) 816:151605. 10.1016/j.scitotenv.2021.151605 34838562

[63] Wang RY Browning M Qin XF He JL Wu WJ Yao Y. Visible green space predicts emotion: evidence from social media and street view data. Appl Geogr. (2022) 148:102803. 10.1016/j.apgeog.2022.102803

[64] Bardhan M Li F Browning M Dong JY Zhang KR Yuan S. From space to street: a systematic review of the associations between visible greenery and bluespace in street view imagery and mental health. Environ Res. (2024) 263:120213. 10.1016/j.envres.2024.120213 39448011

[65] Wang X Guan C. Assessing green space exposure in high density urban areas: a deficiency-sufficiency framework for Shanghai. Ecol Indic. (2025) 175:113494. 10.1016/j.ecolind.2025.113494

[66] Yuen B Hien WN. Resident perceptions and expectations of rooftop gardens in Singapore. Landsc Urban Plann. (2005) 73:263–76. 10.1016/j.landurbplan.2004.08.001

[67] Zheng YC Lin T Hamm NAS Liu J Zhou TY Geng HK. Quantitative evaluation of urban green exposure and its impact on human health: a case study on the 3-30-300 green space rule. Sci Total Environ. (2024) 924:171461. 10.1016/j.scitotenv.2024.171461 38461976

[68] Cheng Y Browning MH Zhao B Qiu B Wang H Zhang J. How can urban green space be planned for a ‘happy city'? Evidence from overhead-to eye-level green exposure metrics. Landsc Urban Plann. (2024) 249:105131. 10.1016/j.landurbplan.2024.105131

[69] Arefi M Aelbrecht P. Urban design and walkability revisited. Urban Design Int. (2023) 28:1–2. 10.1057/s41289-023-00214-338673315

[70] Forsyth A. What is a walkable place? The walkability debate in urban design. Urban Design Int. (2015) 20:274–92. 10.1057/udi.2015.22

[71] Guo ZW Luo KQ Yan ZX Hu A Wang CS Mao Y. Assessment of the street space quality in the metro station areas at different spatial scales and its impact on the urban vitality. Front Archit Res. (2024) 13:1270–87. 10.1016/j.foar.2024.06.006

[72] Lyu X He YY Yin S Wong SM Tse TKT Ng E. Evaluating urban design strategies for pedestrian-level ventilation improvement in a high-density urban living environment - a LiDAR and wind tunnel study. Build Environ. (2025) 269:112439. 10.1016/j.buildenv.2024.112439

[73] Jim CY. Sustainable urban greening strategies for compact cities in developing and developed economies. Urban Ecosyst. (2013) 16:741–61. 10.1007/s11252-012-0268-x39093393

[74] Boeri A Longo D Orlandi S Roversi R Turci G. Community engagement and greening strategies as enabling practices for inclusive and resilient cities. Int J Environ Impact. (2022) 5:1–14. 10.2495/EI-V5-N1-1-14

[75] Bressane A Loureiro AIS Medeiros LCD Negri RG Goulart APG. Overcoming barriers to managing urban green spaces in metropolitan areas: prospects from a case study in an emerging economy. Sustainability. (2024) 16:7019. 10.3390/su16167019

[76] Masterson VA Stedman RC Enqvist J Tengö M Giusti M Wahl D. The contribution of sense of place to social-ecological systems research: a review and research agenda. Ecol Soc. (2017) 22:49. 10.5751/ES-08872-22014930174746

[77] Shao D Zoh K Xie YZ. The spatial differentiation mechanism of intangible cultural heritage and its integration with tourism development based on explainable machine learning and coupled coordination models: a case study of the Jiang-Zhe-Hu in China. Herit Sci. (2024) 12:414. 10.1186/s40494-024-01528-3

[78] Zhu W He W Li QS. Hybrid AI and big data solutions for dynamic urban planning and smart city optimization. IEEE Access. (2024) 12:189994–90006. 10.1109/ACCESS.2024.3516544

[79] Bibri SE. Data-driven smart sustainable cities of the future: urban computing and intelligence for strategic, short-term, and joined-up planning. Comput Urban Sci. (2021) 1:8. 10.1007/s43762-021-00008-9

[80] Son TH Weedon Z Yigitcanlar T Sanchez T Corchado JM Mehmood R. Algorithmic urban planning for smart and sustainable development: systematic review of the literature. Sustain Cities Soc. (2023) 94:104562. 10.1016/j.scs.2023.104562

[81] Li LH Lin XH Yang XT Luo ZW Wang M. Digital governance and urban government service spaces: understanding resident interaction and perception in Chinese cities. Land. (2024) 13:1403. 10.3390/land13091403

[82] Li W Zhang J Guo XJ Zhou Y Yang F Li RL. Digitally driven urban governance: framework and evaluation in China. Sustainability. (2024) 16:9673. 10.3390/su16229673

[83] Wilson B Neale C Roe J. Urban green space access, social cohesion, and mental health outcomes before and during Covid-19. Cities. (2024) 152:105173. 10.1016/j.cities.2024.105173

[84] Lee KO Mai KM Park S. Green space accessibility helps buffer declined mental health during the COVID-19 pandemic: evidence from big data in the United Kingdom. Nat Mental Health. (2023) 1:124–34. 10.1038/s44220-023-00018-y

[85] Chen YR Nakagomi A Hanazato M Abe N Ide K Kondo K. Perceived urban environment elements associated with momentary and long-term well-being: an experience sampling method approach. Sci Rep. (2025) 15:4422. 10.1038/s41598-025-88349-x 39910180 PMC11799535

[86] Wang RY Helbich M Yao Y Zhang JB Liu PH Yuan Y. Urban greenery and mental wellbeing in adults: cross-sectional mediation analyses on multiple pathways across different greenery measures. Environ Res. (2019) 176:108535. 10.1016/j.envres.2019.108535 31260914

[87] Lei BH Li N Guo Y Wang ZH Wei JY Chen RZ. Rapid data collection and processing in dense urban edge computing networks with drone assistance. Phys Commun. (2024) 66:102462. 10.1016/j.phycom.2024.102462

[88] Wang C Peng GH De Baets B. Joint global metric learning and local manifold preservation for scene recognition. Inf Sci. (2022) 610:938–56. 10.1016/j.ins.2022.07.188

